# Identification and Validation of Potential New Biomarkers for Prostate Cancer Diagnosis and Prognosis Using 2D-DIGE and MS

**DOI:** 10.1155/2015/454256

**Published:** 2015-01-15

**Authors:** Cordelia Geisler, Nadine T. Gaisa, David Pfister, Susanne Fuessel, Glen Kristiansen, Till Braunschweig, Sonja Gostek, Birte Beine, Hanna C. Diehl, Angela M. Jackson, Christoph H. Borchers, Axel Heidenreich, Helmut E. Meyer, Ruth Knüchel, Corinna Henkel

**Affiliations:** ^1^Institute of Pathology, RWTH Aachen University, 52074 Aachen, Germany; ^2^Department of Urology, RWTH Aachen University, 52074 Aachen, Germany; ^3^Department of Urology, University Hospital Carl Gustav Carus, 01307 Dresden, Germany; ^4^Institute of Pathology, University Hospital Bonn (UKB), 53127 Bonn, Germany; ^5^Medizinisches Proteom-Center, Ruhr-University Bochum, 44801 Bochum, Germany; ^6^Leibniz-Institut für Analytische Wissenschaften ISAS e.V., 44139 Dortmund, Germany; ^7^University of Victoria-Genome British Columbia Proteomics Centre, University of Victoria, Victoria, BC, Canada V8Z 7X8; ^8^Department of Biochemistry and Microbiology, University of Victoria, Victoria, BC, Canada V8W 2Y2

## Abstract

This study was designed to identify and validate potential new biomarkers for prostate cancer and to distinguish patients with and without biochemical relapse. Prostate tissue samples analyzed by 2D-DIGE (two-dimensional difference in gel electrophoresis) and mass spectrometry (MS) revealed downregulation of secernin-1 (*P* < 0.044) in prostate cancer, while vinculin showed significant upregulation (*P* < 0.001). Secernin-1 overexpression in prostate tissue was validated using Western blot and immunohistochemistry while vinculin expression was validated using immunohistochemistry. These findings indicate that secernin-1 and vinculin are potential new tissue biomarkers for prostate cancer diagnosis and prognosis, respectively. For validation, protein levels in urine were also examined by Western blot analysis. Urinary vinculin levels in prostate cancer patients were significantly higher than in urine from nontumor patients (*P* = 0.006). Using multiple reaction monitoring-MS (MRM-MS) analysis, prostatic acid phosphatase (PAP) showed significant higher levels in the urine of prostate cancer patients compared to controls (*P* = 0.012), while galectin-3 showed significant lower levels in the urine of prostate cancer patients with biochemical relapse, compared to those without relapse (*P* = 0.017). Three proteins were successfully differentiated between patients with and without prostate cancer and patients with and without relapse by using MRM. Thus, this technique shows promise for implementation as a noninvasive clinical diagnostic technique.

## 1. Introduction

Prostate cancer is the most commonly occurring cancer among men in economically developed countries. In 2008, 62 out of 100,000 men were diagnosed with the disease [1]. Worldwide, 248,500 men died of prostate cancer in 2008 [1], although most men diagnosed with prostate cancer die from causes other than prostate cancer [2]. Some prostate cancers are clinically relevant from the start, while others will acquire clinical significance over the years [3, 4]. High-grade prostatic intraepithelial neoplasia often develops into prostate cancer [5–7], although many prostate cancers may remain indolent for 10–15 years or longer [8]. Today, the “gold standard” for the treatment of prostate cancer is prostatectomy, but approximately one-third of all prostatectomy patients will develop a “biochemical relapse” [9, 10], which is defined as the elevation of prostate specific antigen (PSA). Almost 100% of patients who show a biochemical relapse will later develop a clinical relapse [11], with metastasis ultimately causing death [12, 13].

Today, prostate cancer is most often diagnosed through positive palpatory findings within a digital rectal examination and/or a high PSA value during PSA-screening; although specificity is low [14–16], histopathological findings from punch biopsies are used for verification. These findings together with clinical data are used for prognosis using so called “nomograms” [17], whereas the accuracy is only 70% [18, 19]. Even postoperative nomograms have accuracies of only 75% [18, 19].

PSA is a suitable biomarker to identify recurrent prostate cancer subsequent to treatment. However, PSA remains questionable as a diagnostic and prognostic marker [20–23], because specificity and sensitivity are low for the current diagnostic cutoff levels of 4 ng/mL [24]. Unfortunately, high levels of blood PSA (>4 ng/mL) are not necessarily caused by the presence of prostate cancer [24]. PSA can be elevated due to inflammation, benign prostate hyperplasia (BPH), and/or infections [25–27]. Moreover, 70% of patients with PSA > 4 ng/mL and <10 ng/mL do not actually have prostate cancer, while 5% with PSA < 0.5 ng/mL actually do have prostate cancer [24]. On the other hand, patients who are diagnosed with prostate cancer are often overtreated [28], as many prostate cancers are indolent, and because reliable biomarkers for the aggressive form of the disease are currently not available. Thus, new biomarkers are urgently needed.

Proteomic approaches are very promising for the discovery of new biomarkers (as reviewed in [29]). 2D-DIGE (two-dimensional difference gel electrophoresis) is an accurate method for the relative quantitation of human proteins, as this technique reduces intergel variability and simplifies gel analysis of small sample amounts [30, 31].

Unfortunately, despite intense research, no clinical biomarker panel for recurrent prostate cancer is available yet as most published biomarkers for prostate cancer are limited to the discovery phase, are still waiting for validation, or could not be validated in independent studies [32]. A huge problem is the availability of prostate cancer patients' tissue. Many prostate cancer biomarker studies used suboptimal sample sets where samples in the study groups were not matched to age, stage, or grade, tissues were not dissected into tumor and tumor-free tissue, or there were not enough followup data available. As an example Pang et al. analyzed lymph node metastatic prostate cancer and benign prostate cancer tissue using 2D-DIGE and MALDI-TOF/TOF-MS to identify potential new biomarker candidates for lymph node metastatic prostate cancer [33]. Unfortunately, the sample sets were not matched with regard to patients' age, tumor stage, and tumor grade. Other studies are working with tissue samples of patients, which already have metastasis at the time of biopsy [34, 35]. Unfortunately, comparison of those retrospective samples does not forcibly lead to biomarkers which are useful to stratify patients without recurrence at the time of diagnosis. Further limitations of publicized studies are the use of a 2D-DIGE minimal labeling system (e.g., [33, 36]), which is not suitable for the detection of proteins with low abundance. Therefore, in the present study, a 2D-DIGE saturation labeling system was used, allowing labeling of 1.000–5.000 cells [37, 38] or 0,5 fmol protein [39] whereby this sensitivity could not be reached by other techniques so far [40].

Multiple reaction monitoring (MRM) is a mass spectrometry technique that provides accurate absolute quantitation of selected proteotryptic peptides [41]. For the most accurate quantitation, a synthetic stable isotope-labelled (SIS) peptide at a known concentration is spiked into the sample. Quantitation of the natural peptide takes place through comparison of the peaks from the natural and the chemically identical SIS-peptides. MRM has been shown to fulfill the requirements needed for the verification of biomarker candidates, as it has the capability to quantify proteins consistently, simultaneously, accurately, and reproducibly in complex samples [41]. Compared to ELISA, lead time is shorter and costs are reduced [41]. As an example, Percy et al. and Domanski et al. have developed multiplexed MRM-based assays for the quantitation of cardiovascular disease biomarkers and cancer biomarkers in human plasma [42, 43]. Until now, these assays are developed to fulfill the requirements for preclinical application for evaluating potential useful biomarkers [42]. But hopefully, MRM-based methods for quantitation of cancer-related protein biomarkers will soon be approved by the US FDA [42], moving this technique one step closer to clinical application [44].

In the present study, patients sample sets for both analyzed patient groups (patients with biochemical relapse versus patients without biochemical relapse) were matched with regard to age, tumor stage, and tumor grade as far as possible. Additionally, manual microdissection of the tissue ensures that the percentage of tumor glands in the analyzed tissue were >80%.

**Figure 1:**
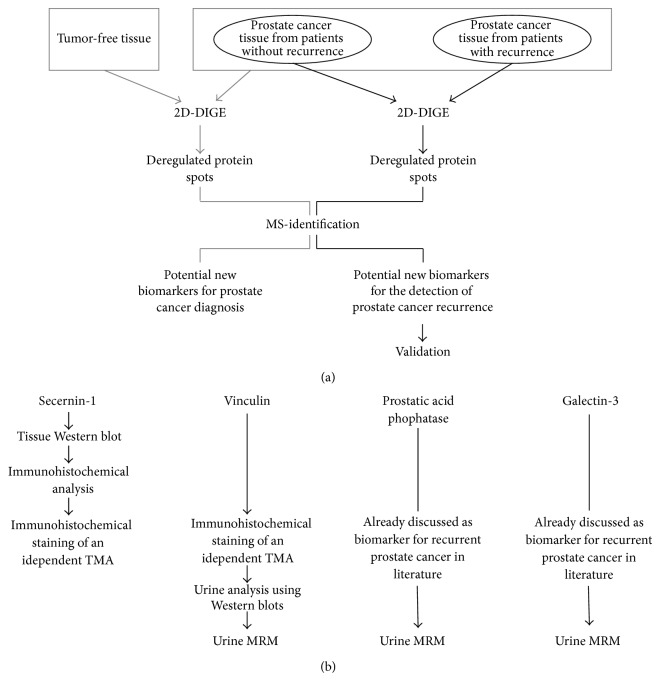
Study design and workflow of prostate cancer biomarker candidate identification (a) and validation (b). (a) Prostate cancer tissue from patients with and without recurrence as well as tumor-free tissue was analyzed using two-dimensional differences in gel electrophoresis (2D-DIGE) and mass spectrometry (MS). (b) Identified potential new biomarker candidates were validated using Western blots, immunohistochemistry, tissue microarrays (TMA), and multiple reaction monitoring (MRM).

Potential new prostate cancer biomarkers were found in a 2D-DIGE study of prostate cancer tissues from patients with and without relapse, with tumor-free tissue samples as controls. The deregulated proteins were identified using mass spectrometry (MS). Ingenuity pathway analyses were accomplished in order to perform functional analysis of the identified proteins. Promising potential biomarker candidates were chosen for further validation with immunohistochemical staining of an independent tissue microarray, Western blots of tissue and urine proteins, and MRM-MS analysis of patients' urine. The detailed study design is shown in Figure 1.

## 2. Material and Methods

### 2.1. Analysis of Tissue Samples

#### 2.1.1. Clinical Specimens

Twelve cancer samples from prostatectomy specimens without relapse, 11 cancer samples with relapse, and 14 tumor free prostate samples corresponding to the tumor samples were analyzed with 2D-DIGE. The same samples were used for Western blot analysis. Where possible, matched patient samples with respect to age, tumor grade, and Gleason score were used for both tumor patient groups (with* versus* without relapse). Only patients without hormonal therapy prior to prostatectomy were included in the study. Samples were obtained from patients treated at the Departments of Urology at the University Hospitals Dresden and Aachen between 1998 and 2010. The study was approved by the local ethics committee (ethics approval Aachen: EK 206/09 and ethics approval Dresden: EK194092004 and EK195092004). Written informed consent was obtained for all specimens. Samples were snap-frozen in liquid nitrogen, and the classification of tumors was done by pathologists in accordance with the UICC TNM System [45].

For details see Table 1. Due to sample limitations, Western blot validation could not always be performed with the identical sample set. Details are listed in Supplementary Table 2  available online at http://dx.doi.org/10.1155/2014/454256.

Validation of potential prostate cancer biomarker candidates by immunohistochemical analysis was done with samples obtained from the Department of Urology in Dresden. Samples were formalin-fixed and paraffin-embedded at the Department of Urology in Dresden. Detailed patient information is listed in Supplementary Table 3.

For validation of an independent sample set, tissue microarrays (TMA) were obtained from the Institute of Pathology, University Hospital Bonn. The study was approved by the Institutional Review Board (IRB) at the University Hospital Bonn, and the IRB waived the need for written informed consent of the participants. Patients underwent surgery between 2004 and 2007 at the University Hospital Bonn and TMA preparation was done as previously described [46, 47]. Detailed patient information is listed in Supplementary Table 4 and Supplementary Table 5.

Samples for Western blot analysis of vinculin in urine were obtained from the University Hospital Aachen. Detailed patient information is listed in Supplementary Table 6.

For the MRM-MS analysis, urine samples from the University Hospital Aachen were used (ethics approval Aachen: EK 206/09). Urine samples were obtained between 2005 and 2010 from patients treated at the Department of Urology, University Hospital Aachen. Samples were snap-frozen in liquid nitrogen and stored at −80°C at the Institute of Pathology, University Hospital Aachen, until use. Only samples from patients without neoadjuvant therapy were included in this study. Detailed information is listed in Supplementary Table 7.

Detailed information which sample set was used for which experiment is listed in Table 1.

#### 2.1.2. Manual Microdissection and Tissue Preparation for 2D-DIGE and Western Blot Analysis

All tissue samples were stored at −80°C prior to protein isolation. Proteins for 2D-DIGE analysis were isolated from 4 mm^2^ of a 14 *μ*m thin cryoconserved section with a minimum of 80% of prostatic glands.* TissueTec* from the embedding and freezing process was removed using 70% ethanol. The sample sections were stained in a series of ultrapure water, haematoxylin, ultrapure water, and 70% ethanol. All liquids were used with* Complete Protease Inhibitor Cocktail Tablets* (Roche, Mannheim, Germany). The areas of interest were marked using the PALM Axiovert 200M (Carl Zeiss Microscopy, Göttingen, Germany) laser and were manually microdissected. Proteins were dissolved in 10 *μ*L lysis buffer (30 mM Tris-HCl, 2 M thiourea, 7 M urea, 4% (w/v) CHAPS; pH 8.0). The extracts were sonicated on ice and centrifuged at 4°C for 15 min and 16,000 ×g. Supernatants were stored at −80°C.

#### 2.1.3. Protein Labeling and Two-Dimensional Difference in Gel Electrophoresis (2D-DIGE)

Protein lysates were labeled with 2 mM Cy5 dye using the GE CyDye DIGE Fluor Labeling Kit (GE Healthcare, UK) according to the manufacturer's instructions. As an internal standard, proteins from all patient samples were pooled and 5 *μ*g were labeled with 2 mM Cy3 dye. Labeled samples were combined. Rehydration buffer (7 mM urea, 2 M thiourea, 2% (w/v) CHAPS, 1% DTT, 1% IPG buffer pH 3-11 NL (GE Healthcare, UK), 0.002% bromphenol blue) was added to give a total volume of 450 *μ*L. Rehydration, isoelectric focusing and gel electrophoresis were performed as described by Labbus et al. [48].

#### 2.1.4. Gel Image Analysis

2D-DIGE gels were visualized using a Typhoon 9410 fluorescence scanner (GE Healthcare) with excitation/emission at 554/575 nm (Cy3) and 648/663 nm (Cy5). Scanning resolution was 100 microns and the photomultiplier tube was set to 550 V. Gel image and statistical analyses were done using the Delta2D 4.0 Software (Decodon, Greifswald, Germany). The Delta2D data set was first normalized by dividing each spot volume by the sum of all spot volumes on the respective gel image. By opening the analysis tool of Delta2D logarithmic function is performed automatically; furthermore data is standardized (resulting in means of zero and standard deviations of one). The recommended workflow includes fusing all images and detecting the spots on the resulting fused image, which contains all spots of the original images. The spot pattern is then transferred to all original images. Therefore, in this approach, no missing values appear. Additionally detailed Delta2D workflow information is described by Berth et al. [49]. Spots showing a quantitative difference of a ≥1.5-fold change between nontumor and tumor groups and between the two tumor groups (i.e., with or* versus* without a relapse), respectively, were included in further analyses. Additionally, either a Student's *t*-test (as a parametric test) or a Mann-Whitney *U*-test (as a nonparametric test) with a *P* value of <0.05 was accepted as statistical relevant. The *U*-test and *t*-test were used because of uncertainty concerning presence of normal distribution (*t*-test [50]; *U*-test [51, 52]).

#### 2.1.5. Protein Identification Using MALDI-TOF MS/MS and LC-MS/MS

Trypsin digestion and protein identification using MALDI-TOF MS and MS/MS were done as previously described [48]. All 2D-DIGE protein spots that were not identified using MALDI-TOF MS were further analyzed using LC-MS/MS as follows. Trypsin-digested proteins were extracted from the gel spot using 10–20 *μ*L extraction solution (0.1% TFA/Acetonitrile 1 : 1) and sonicated on ice for 15 min. The supernatant containing the extracted peptides was transferred to a new glass tube. For a second extraction step, the gel spot was once more incubated and sonicated with 10–20 *μ*L extraction solution for 15 min. The supernatants were combined and remaining acetonitrile was removed in a vacuum Speedvac concentrator 5301 (Eppendorf, Germany). Peptides were diluted with 0.1% TFA to a final volume of 17 *μ*L. Peptide concentration was determined by amino acid analysis as described elsewhere [53]. Mass spectrometric analysis was done using a LTQ Orbitrap Velos (Thermo Scientific, San Jose, USA) online, coupled to an Ultimate 3000 RSLCnano system (Dionex, Idstein, Germany). Samples were preconcentrated on a trap column (Acclaim PepMap 100, 300 *μ*m × 5 mm, C18, 5 *μ*m, and 100 Å) and separated on an Acclaim PepMap 100, 75 *μ*m × 25 cm, C18, 3 *μ*m, and 100 Å analytical column. The flow rate was 0.4 *μ*L/min with a linear gradient of 4–35% buffer B (84% acetonitrile and 0.1% formic acid) for 65 min. MS-analyses were done in FT-master scan mode. The collision energy was 35 eV with an activation time of 10 seconds. The intensity counts for MS/MS were set to 500 counts with a dynamic exclusion time of 35 seconds. Five most intense precursor ions were selected for fractionation in a data-dependent acquisition approach (TOP5). Columns were washed after each sample. Protein identification was achieved using Proteome Discoverer 1.3 (Version 1.3.0.399; Thermo Scientific, Bremen) with Mascot database (Version 2.3) as search engine and UniprotKB/Swiss-ProtDatabase (Uniprot/Swissprot-Release 2012_02; 534.695 entries) with the following search criteria: protease trypsin, one missed cleavage, 400–10,000* m/z*, 1.5 signal-to-noise threshold, mass tolerance of 5 ppm, and a fragment and precursor mass tolerance of 0.4 Da. FDR (false discovery rate) were calculated using the proteome discoverer application's decoy database search feature (Reference: Xcalibur Proteome Discoverer Version 1.1 User Guide XCALI-97276 Revision A October 2009, http://sjsupport.thermofinnigan.com/TechPubs/manuals/Discoverer_UG.pdf), and the FDR was set to a threshold of 0.01. A decoy approach was used for identification. The used protein inference algorithm was used as stated in the Mascot Manual (http://www.matrixscience.com/help/interpretation_help.html#GROUPING): First, Mascot takes the protein with the highest protein score and calls this hit number 1. Then it takes all other proteins that share the same set of peptide matches or a subset and includes these in the same hit. In the report, they are listed as same-set and subset proteins. With these proteins removed from the list, Mascot now takes the remaining protein with the highest score and repeats the process until all the significant peptide matches are accounted for (Mascot Manual, http://www.matrixscience.com/help/interpretation_help.html#GROUPING,  paragraph “Protein inference”). Protein identification relied on proteins and unique peptides. For more sensitive analysis, an LTQ Velos Pro (Thermo Scientific, San Jose, USA) was used. The instrument was online coupled to an Ultimate 3000 RSLCnano System (Dionex, Idstein, Germany) equipped with an Acclaim PepMap RSLC, 75 *μ*m × 25 cm, C18, 2 *μ*m, and 100 Å column. All LC and analysis methods remained constant between the two MS platforms.

Ten proteins (Supplementary Table 12 and Supplementary Table 13) were identified using the Maxis 4G (Bruker Daltonik, Bremen, Germany) controlled by Compass 1.3 for micrOTOF-SR1 Software (Bruker Daltonik, Bremen, Germany). The MS instrument was online coupled to an U3000 LC system (Dionex, Idstein, Germany), controlled by* Chromeleon 6.8 SR8*, and equipped with a 25 cm long C18 analytical column (ID 75 *μ*m) heated up to 50°C. Thirty *μ*L of each sample in 0.1% trifluoroacetic acid was injected and analyzed at a flow rate of 350 nL/min with a linear gradient of 5 to 40% acetonitrile achieved through dilution with buffer B (84% acetonitrile and 0.1% formic acid). Capillary voltage was 4800 and flow of dry gas was 4 L/min. Protein identification was performed using ProteinScape 2.0 (Bruker Daltonic, Bremen, Germany) with Mascot (Version 2.3) as search engine and UniprotKB/Swiss-Prot Database (Uniprot/Swissprot-Release 2012_02; 534.695 entries) with the following search criteria: protease trypsin and one missed cleavage, and variable methionine oxidation was allowed. The mass tolerance was 15 ppm for peptides and 0.1 Da for MS/MS identification. FDR and protein inference were calculated as described above.

#### 2.1.6. Ingenuity Pathway Analysis

Ingenuity pathway analysis (IPA, QIAGEN Redwood City, http://www.qiagen.com/ingenuity) was used to determine* Top Diseases*,* Biofunctions*, and* Localization* of the identified proteins. Direct and indirect relationships were included in the analysis. Molecules and relationships were considered as long as the species was human and molecules and the relationships were experimentally observed. The number of molecules for type, localization, molecular, and cellular functions, as well as the role of the identified proteins in development and function of the physiological systems, were counted.

#### 2.1.7. Protein Selection for Further Validation

Based on IPA and—more importantly—on literature review we selected four proteins for further validation using Western blot analysis, immunohistochemical analysis, and/or MRM analysis. For study design, see Figure 1.

#### 2.1.8. Bradford-Assay

Unless otherwise specified, protein concentrations were determined using the Bio-Rad Bradford assay (Bio-Rad Laboratories, Hercules/California, USA). Forty *μ*L of ultrapure water was mixed with 10 *μ*L Bradford reagent and 1 *μ*L protein sample. Forty *μ*L ultrapure water mixed with 10 *μ*L Bradford reagent was used as a blank. Samples were measured with an ELISA Reader Infinite M200 (Tecan, Männedorf, Switzerland) at an extinction of 595 nm. Protein concentrations were determined by comparing the absorption at 595 nm with dilution series consisting of 10 *μ*L Bradford reagent and 1 *μ*g, 2 *μ*g, 3 *μ*g, 4 *μ*g, or 5 *μ*g of bovine serum albumin (BSA), respectively, filled up to 40 *μ*L ultrapure water.

#### 2.1.9. Western Blot

Ten *μ*g protein samples were mixed 1 : 4 (v : v) with SDS sample buffer (4% SDS, 0.5 M Tris-HCl pH 6.8, 40% glycerol, 10% *β*-mercaptoethanol, and 0.002% bromophenol blue). Samples were incubated for 5 min at 95°C and loaded onto a Novex NuPAGE 4–12% Bis-Tris gel (Invitrogen, Carlsbad, CA, USA). After electrophoresis at 130 V until the bromophenol blue front reached the end of the gel, proteins were electrotransferred onto polyvinylidene fluoride membranes (Millipore Corporation, Bedford, MA, USA). Blots were blocked with 10% milk powder in TBS-T (0.5 M NaCl, 1 M tris pH 7.5, 0.5% tween). Antibodies used for immunodetection of desired proteins are listed in Table 2. For visualization, the membrane was incubated with SuperSignal West Femto Maximum Sensitivity Substrate (Thermo Scientific, Rockford, IL, USA) and exposed to Amersham Hyperfilm (GE Healthcare). Densitometric analyses of the results were performed using ImageJ 1.45 (Oracle Corporation, National Institute of Health, USA).

#### 2.1.10. Immunohistochemistry

For immunohistochemistry, FFPE tissues were dewaxed 3 times with xylene (15 min each), 2 times with 100% ethanol (10 min each), 2 times with 96% ethanol (5 min each) and 70% ethanol (5 min each), and 3 times with ultrapure water (5 min each). For antigen retrieval, slides were incubated in 20% citrate buffer pH 6.0 for 30 min in a 98°C water bath and then cooled for 30 min and washed 5 times with phosphate buffered saline (PBS). Blocking of endogen peroxidase was done with 3% H_2_O_2_ for 15 min. Slides were washed 5 times with PBS prior to blocking of unspecific antibody binding with DAKO protein block serum-free (Dako, Hamburg, Germany) for 20 min at 37°C. Primary antibodies were diluted in 1% milk powder in PBS. The incubation conditions are listed in Table 3.

Slides were washed with PBS and incubated at 37°C with DAKO Envision premade biotin-free enhancer solution (for detection of mouse and rabbit primary antibodies) for 3 min and then were washed and incubated in PBS for one hour. A DAKO Liquid DAB Substrate Chromogen System Kit (DAKO) was used for development: 1 mL buffer was mixed with 20 *μ*L DAB. Slides were incubated with this solution for 5 to 10 min and then were washed with ultrapure water and incubated in PBS for 5 min. Incubation in hematoxylin for 10 min was used for counterstaining. Slides were incubated in tap water for 10 min and dehydrated once in 70% ethanol for 1 min, once in 96% ethanol for 1 min, twice in 100% ethanol for 2 min, and twice in xylene for 5 min. The stained tissue samples were mounted with vitroclud (R. Langenbrinck, Germany) and glass cover slides in case the Remmele score were used for scoring of immunohistochemical stainings. Scoring was done as described elsewhere [54] whereby the described scoring of nuclear staining was adapted to the cytoplasmatic staining (adapted Remmele score).

### 2.2. Analysis of Urine Samples

#### 2.2.1. Preparation of Urine Samples for Vinculin Western Blot Analysis

Urine proteins were precipitated with 10 volumes of ice cold methanol. Samples were incubated for 30 min at −20°C followed by centrifugation at 16,000 ×g at 4°C for 20 min. The supernatant was discarded and the sediment was dried at room temperature. Proteins were suspended in 10 *μ*L lysis buffer (30 mM Tris-HCl, 2 M thiourea, 7 M urea, and 4% (w/v) CHAPS; pH 8.0).

#### 2.2.2. Preparation of Urine Samples for MRM-MS Analysis

A total of 23 urine samples from the University Hospital Aachen were used for MRM analysis. All samples were prepared as follows: 2 mL urine, corresponding to 160 *μ*g of protein, was centrifuged at 3,900 ×g for 30 min at 4°C to remove cells and cell debris. Each supernatant was transferred to a Millipore Amicon Ultra-4 centrifugal filter unit (10,000 MWCO (molecular weight cutoff)) and centrifuged at 3,900 ×g until concentrated 4-fold. The concentrated protein solution was washed on filter with 4 mL 20% acetonitrile and 25 mM ammonium bicarbonate and centrifuged at 3,900 ×g until the volume was reduced to 500 *μ*L. In a second washing step, the protein solution was diluted with 2 mL of 25 mM ammonium bicarbonate and centrifuged at 3,900 ×g until the volume was reduced to 100 *μ*L. A 18.75 *μ*L sample was removed and denatured with 81.25 *μ*L of 8 M Urea containing 0.1 M ammonium bicarbonate.

Trypsin digestion was based on the protocol by Selevsek et al. [55]. Briefly, 100 *μ*L of the concentrated and denatured protein solution was reduced with 1.2 M DTT (final concentration of 12 mM) for 30 min at 37°C. Proteins were alkylated with 0.5 M iodoacetamide (final concentration of 40 mM) and incubated for 30 min at 37°C. The samples were diluted with 0.1 M ammonium bicarbonate (Sigma-Aldrich, USA) to a final urea-concentration below 2 M. The proteins were then digested with Worthington TPCK trypsin (0.9 mg trypsin in 1 mL 10 mM CaCl_2_-dihydrate containing 25 mM ammonium bicarbonate) at a 20 : 1 (protein to enzyme) ratio and incubated at 37°C overnight.

#### 2.2.3. Development of the MRM Assay

The development of an MRM assay involves several stages, the first being the selection of the target peptides that will represent each target protein. The selection rules for these peptides has been discussed in several previous papers [42] and will not be repeated here. Briefly, peptide selection involves optimizing the peptide mass spectrometric detectability by taking into account factors such as the peptide length, the absence of oxidizable residues, and other factors such as the avoidance of residue combinations such as RK and KK, which can lead to missed cleavages. These are avoided because they could lead to a reduction in sensitivity by multiple isoforms of the target peptides. The efficiency of tryptic digestion <95% was verified with ExPASy Peptide-Cutter (http://web.expasy.org/peptide_cutter/). If all of the above criteria were met, peptides were ranked based on their previous detection using both The GPM (http://gpmdb.thegpm.org/index.html) and Peptide Atlas (https://db.systemsbiology.net/sbeams/cgi/PeptideAtlas/Search) databases. All of the SIS-peptides used in this study are listed in Supplementary Table 8.

#### 2.2.4. Synthesis and Purification of Isotopically Labeled Standard Peptides

Synthesis and purification of SIS-peptides were done as previously described [43].

#### 2.2.5. MRM Q1/Q3 Ion Pair Selection Using Direct Infusion (Peptide Optimization)

Prior to MRM analysis, the ion pairs (called “transitions”) for protein quantification had to be selected. This “peptide optimization” was done as previously described [43], with the following changes: the nebulizer gas flow was 60 psi and the scanning time was 500 ms. A list of all possible b- and y-ion series for 2+ and 3+ precursor ion charge spanning a range of* m/z* from 200 to 1100 was generated using the Agilent MassHunter Optimizer For Peptides Software (Version B.05.00, Agilent). Product ions within 1 Da were excluded to ensure that only a single targeted product-ion was measured. All +2 or greater product ions were eliminated from the method using Mathew Monroe's Molecular Weight Calculator Freeware (http://www.alchemistmatt.com/). For each peptide the top 5 transitions, defined as those transitions with the most abundant signals, were selected for chemical interference screening.

#### 2.2.6. Interference Screening of SIS-Peptides in Urine Samples

Interference testing has been described elsewhere for plasma [43], and a similar process is followed for urine. Basically, interference testing requires examining the ratios of each endogenous and SIS-peptide's transitions in buffer and in urine. If there are no interferences, the ratio in buffer and in urine should be the same.

A pooled urine sample from 5 female and 5 male donors collected from first void, midstream, and with sodium azide added to a produce a final concentration of 0.05% (Bioreclamation LLC, Westbury NY, USA; Lot No BRH683580), was used as the matrix for the interference testing.

Urine samples were prepared for tryptic digestion as described above. 100 fmol/*μ*L of each measured SIS-peptide was added to tryptic-digested urine and the samples were desalted on a Waters Positive Pressure Manifold using Waters Oasis 96-well *μ*Elution Plates 30 *μ*g HLBa sorbent (batch number 115B) according to the manufacturer's instructions. Briefly, *μ*Elution plates were activated with 200 *μ*L of 100% methanol and equilibrated with 200 *μ*L 0.15% formic acid. Samples were diluted 1 : 1 with formic acid before adding them to sorbent. The sorbent was washed twice with 200 *μ*L 0.1% formic acid prior to elution with 100 *μ*L of 50% acetonitrile/0.1% formic acid. After a short centrifugation step, samples were frozen at −80°C and lyophilized overnight. Before LC/MRM-MS analysis, samples were rehydrated in 0.1% formic acid (mobile phase A). Both urine digest and matrix-free samples were analyzed in triplicate.

All LC/MRM-MS measurements were carried out on an Agilent 1290 infinity UHPLC system coupled to an Agilent 6490 triple quadruple mass spectrometer (Agilent Technologies, Santa Clara, CA, USA) with MassHunter Workstation Software (Agilent, B.04.01). Twenty *μ*L of each sample was injected and separated at a flow rate of 400 *μ*L/min on an Agilent Zorbax RRHD Eclipse Plus C18, 2.1 × 150 mm and 1.8 *μ*m analytical column using a mobile phase gradient from 3 to 90% phase B (90% acetonitrile/0.1% formic acid) in a 43 min analysis. The gradient was as follows: 0 min, 3% B; 1,5 min, 7% B; 16 min 15% B; 18 min, 15,3% B; 33 min, 25% B; 38 min, 45% B; 29 min, 90% B; 43 min, 3% B. All acquisition methods used have previously described acquisition parameters [43] and scheduled retention times with a minimum dwell time of 20 ms to allow for the maximum number of peptides to be analyzed per injection.

The most intense interference-free signal producing transition was later used for peptide quantification (the quantifier) while other two transitions were used for quality control (the qualifiers). All quantifier and qualifier transitions are listed in Supplementary Table 8.

#### 2.2.7. Concentration Balancing of SIS-Peptides in Urine Samples

For the highest quantitation accuracy, the concentration of each SIS-peptide should match as closely as possible to the concentration in the sample [56]. Abundance of each SIS-peptide should be at least between 1 × 10^3^ and 1 × 10^4^ to ensure optimal peak shape and therefore correct integration. The difference in peak areas between the natural and SIS peaks should be no more than a factor of 10 for optimal quantitation. Supplementary Table 9 shows the final SIS-peptide concentrations.

#### 2.2.8. MRM-Based Quantitation of Vinculin, Galectin-3, and Prostatic Acid Phosphatase in Urine and MRM Data Analysis

Urine samples for scheduled MRM analysis were prepared as described above. Concentration-balanced SIS-peptides were spiked in (Supplementary Table 9) and the samples were desalted and lyophilized and then reconstituted in 20 *μ*L 0.1% formic acid prior to analysis as described in Interference Screening of SIS-Peptides in Urine Samples section.

MRM data was processed and evaluated with Agilent's MassHunter Quantitative Analysis Software (Agilent B.04.00) and Agilent's Integrator Algorithm for Peak Integration. All peaks were verified for correct chromatographic peak selection and integration. The ratio between the natural peptide peak area and SIS-peptide's peak area was calculated as the response ratio (RR). Natural peptide concentrations were calculated by multiplication of the RR by the concentration of the SIS-peptides that had been spiked into the sample. The accuracy of the calculation was further increased by verifying the purity of the SIS-peptides by amino acid analysis (AAA) and capillary zone electrophoresis (CZE); data are listed in Supplementary Table 9.

#### 2.2.9. Statistical Evaluation

Statistical analyses for Western blot analysis, immunohistochemical analysis, and MRM analysis were done using SPSS 15.0 (SPSS, Chicago, IL). *P* values of <0.05 were defined as statistically significant. Two-sided Mann-Whitney *U*-test was used to detect differences in abundance levels among the various groups studied, based on the Western blot, immunohistochemical, and MRM-MS results.

## 3. Results

### 3.1. Identification of Novel Potential Biomarkers for Prostate Cancer in Tissue Using 2D-DIGE with MS Identification

**Figure 2:**
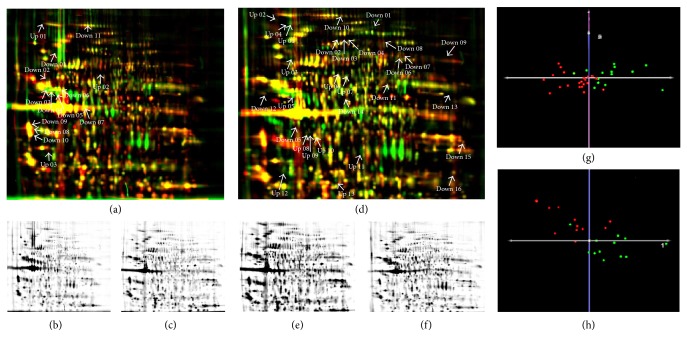
2D-DIGE analysis of 14 tumor free prostate tissue samples, 12 prostatectomy samples from prostate cancer patients without relapse in a 5-year followup, and 11 prostatectomy samples from patients with relapse. (a) Overlay of 2D-DIGE gels of prostatectomy samples from tumor-free tissue areas ((b), green) and patients with prostate cancer ((c), red). (d) Overlay of 2D-DIGE gels from prostatectomy samples from patients without ((e), red) and with ((f), green) relapse. Downregulated spots in prostate cancer and prostate cancer with relapse, respectively, are annotated with “down.” Upregulated spots in these samples are annotated with “up.” Principal component analysis (PCA) of prostate cancer (red) and tumor-free tissue (green) (g) and prostate cancer samples without (red) and with relapse (green) (h).

For the identification of differentially regulated proteins in prostate cancer, 12 prostatectomy samples from prostate cancer patients without biochemical relapse, 11 prostatectomy samples from patients with biochemical relapse, and 14 corresponding tumor-free prostate cancer tissue samples were comparatively analyzed by 2D-DIGE saturation labeling. Comparison of all samples revealed 1000 gel spots common to all gels by using the Delta2D software. Tumor and tumor-free samples as well as tumor samples from patients with and without biochemical relapse could be distinguished from each other using principal component analysis (Figure 2). Comparison of the tumor-free* versus* the tumor samples revealed 37 protein spots with bigger normalized volume and 27 protein spots with smaller normalized volume in the tumor samples compared to the tumor-free samples (Supplementary Table 10). Of these, 14 protein spots were identified using MALDI-MS and LC-MS/MS (Figure 2, Table 4, and Supplementary Table 12).

In addition, 12 prostatectomy samples from patients without biochemical relapse and 11 prostatectomy samples from patients with biochemical relapse were compared to reveal proteins involved in tumor aggressiveness. The analysis resulted in 22 protein spots which showed bigger normalized volumes and 13 protein spots which showed smaller normalized volumes prostatectomy samples of patients with biochemical relapse compared to samples of patients without biochemical relapse (Supplementary Table 11). Of these, 29 deregulated protein spots were identified using MALDI-MS and LC-MS-MS (Supplementary Table 13). Among these, prostatic acid phosphatase (PAP), vinculin, secernin-1 (SCRN1), lamin A/C, and gelsolin were identified. All of the identified proteins are listed in Table 5.

### 3.2. Ingenuity Pathway Analysis (IPA) of the Identified Proteins

**Figure 3:**
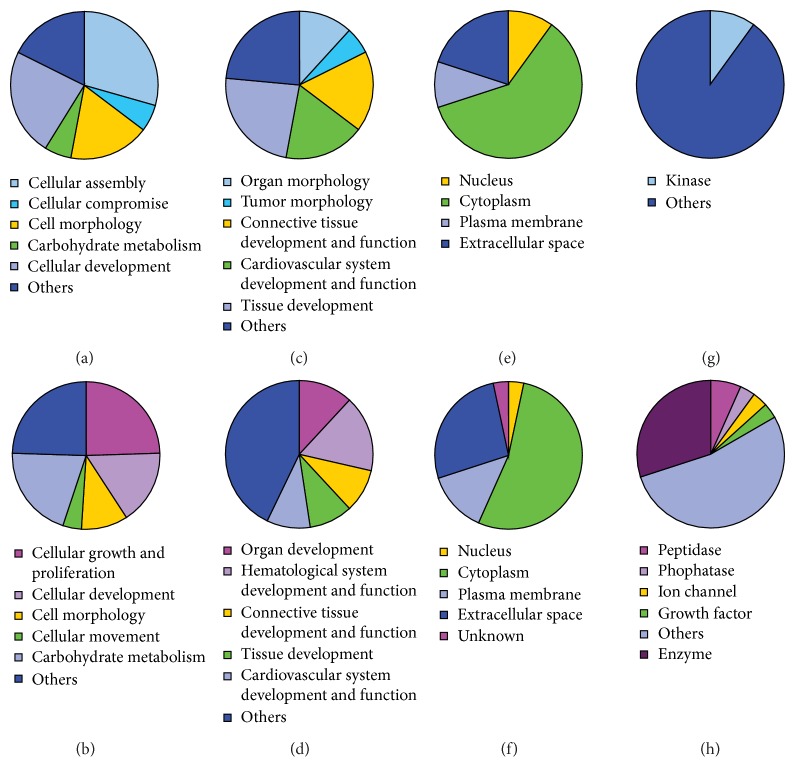
Ingenuity pathway analysis of the proteins that were deregulated between tumor-free samples and prostate cancer samples ((a), (c), (e), and (g)) as well as proteins that were deregulated between prostate cancer samples from patients with and without relapse ((b), (d), (f), and (h)). Distribution of molecular and cellular functions ((a) and (b)), role of the identified proteins in development and function of the physiological systems ((c) and (d)), localisation ((e) and (f)), and type of the identified proteins ((g) and (h)) are shown.

Ingenuity pathway analysis of the potential new biomarkers identified using 2D-DIGE and MS revealed that most of the deregulated proteins are located in the cytoplasm. As shown Figure 3, 60.0% of the differentially-expressed proteins in the tumor* versus* tumor-free sample set, and 53.3% within the aggressive* versus* non aggressive tumor sample set, were located in the cytoplasm. Proteins that are differentially expressed between tumor-free tissue and prostate cancer tissue were mostly associated with cellular assembly (5 proteins), cellular development (4 proteins), cell morphology (3 proteins), cellular compromise (i.e., associated with damage or degeneration of cells; 1 protein), and carbohydrate metabolism (1 protein). The differentially expressed proteins in tumors with or without relapse were mostly associated with cellular growth and proliferation (12 proteins), cellular development (10 proteins), cellular movement (8 proteins), cell morphology (5 proteins), and carbohydrate metabolism (2 proteins). Detailed results, as well as the classification of the identified proteins and their functions in development and in the physiological system, are shown in Table 6 and Figure 3.

### 3.3. Validation of Potential Tissue Biomarker Candidates Found by DIGE

**Figure 4:**
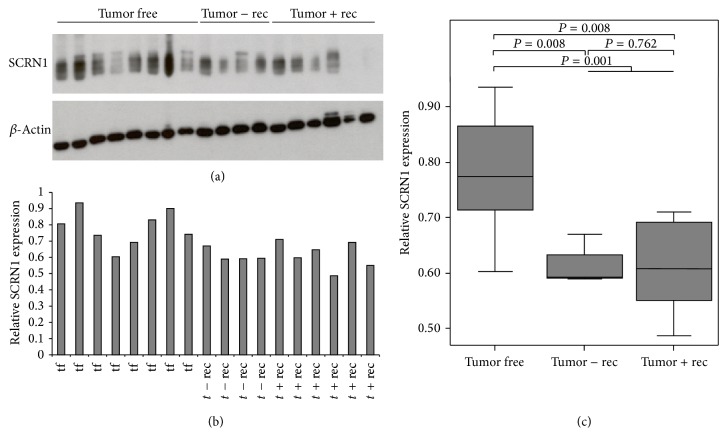
Western blot analysis of secernin-1 (SCRN1) and *β*-actin as a house keeping protein in prostate cancer tissue and tumor-free tissue samples. (a) Western blot analysis. (b) Relative SCRN1 expression levels were calculated densitometrically in reference to the *β*-actin expression level. (c) Boxplot of the densitometrically determined SCRN1 expression levels. A significant difference between tumors without (*t* − rec) and with recurrence (*t* + Rec) is not detectable (*P* = 0,762) but SCRN1 is significantly downregulated in prostate cancer tissue compared to tumor-free tissue samples (tf) (*P* = 0.001).

**Figure 5:**
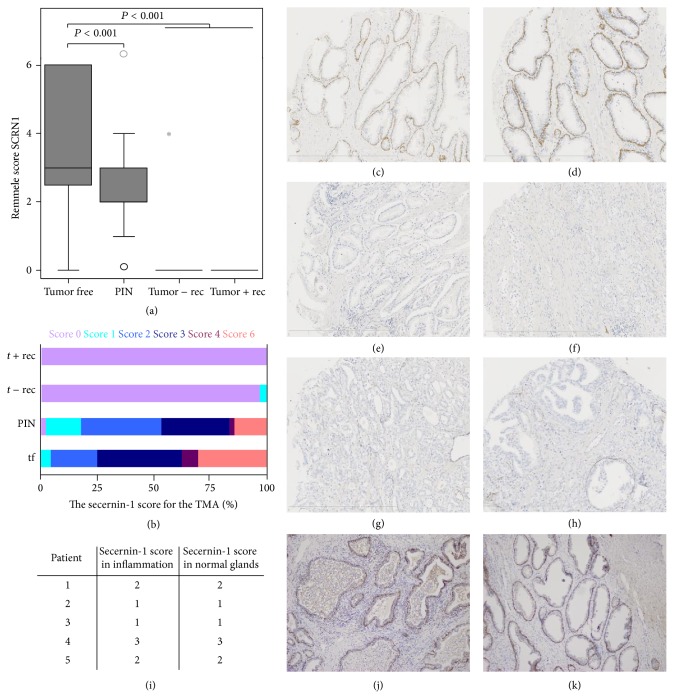
(a)–(h) Representative immunohistochemistry of secernin-1 in an independent tissue microarray (TMA) obtained from the University Hospital Bonn. (a) Boxplot of the secernin-1 expression levels in the analyzed patient groups. (b) Percentages of each score in each analyzed patients group. For more detailed information, an adapted Remmele score was used for classification of the secernin-1 expression. (c) and (d) Tumor-free prostatectomy samples. (e) and (f) Prostatectomy samples of prostate cancer patients without relapse. (g) and (h) Prostatectomy samples of prostate cancer patients with relapse. Secernin-1 expression is significantly downregulated in prostate cancer tissue compared to tumor-free tissue samples (*P* < 0.001). Downregulation of secerin-1 starts in the peri-intraepithelial neoplasia (PIN) as PIN lesions showed less secerin-1 expression than tumor-free tissue samples (*P* < 0.001) but stronger secernin-1 expression than prostate cancer tissue (*P* < 0.001). (i)–(k) Representative immunohistochemical staining of 5 prostatectomy samples of patients with prostatitis (j) and corresponding normal prostate tissue (k) as well as a table (i) of the results of all 5 analyzed patients obtained from the University hospital Aachen. Secernin-1 staining intensity is not affected by inflammation: the five analyzed tumor-free tissue samples showed the same staining intensity for secernin-1 as the corresponding inflamed tissue.

Some candidates found by 2D-DIGE and MS (secernin-1, vinculin, prostatic acid phosphatase (PAP), and galectin-3) were selected for further validation. Two of those, PAP and galectin-3, have already been suggested as potential biomarkers for prostate cancer.* Secernin-1* 2D-DIGE analysis also revealed that secernin-1 shows significantly lower abundance in recurrent prostate cancer tumors compared to prostate cancer tumors without biochemical relapse. For Western blot validation, eight tumor-free tissue samples and four prostatectomy samples from patients without and six prostatectomy samples from patients with relapse were analyzed (Figure 4). Secernin-1 showed a significant downregulation in tumors (*P* = 0.001) but no deregulation between tumors with and without relapse (*P* = 0.762). Further immunohistochemical analysis of the 13 prostatectomy samples from prostate cancer patients without relapse, the 14 samples from patients with relapse, and the 43 tumor free tissue samples (kindly provided by the University Hospital Dresden) validated the results of the Western blot: tumors from patients with and without relapse showed a significantly lower immunohistochemical score (median 1.0 for tumor tissue) than tumor-free tissue (mean 3.00; *P* < 0.001; Supplementary Figure 1). Differences in expression levels between tumors with and without relapse could not be shown. Moreover, immunohistochemical staining showed secernin-1 expression in the basal cell layer but not in the luminal cells itself. To further examine the potential use of secernin-1 as a potential biomarker candidate for prostate cancer, and to discriminate between prostate cancer and prostatitis, five tissues with prostatitis were analyzed. In these experiments, the areas with prostatitis showed no difference in secernin-1 expression levels compared to the noninflamed, tumor-free tissue areas (Figures 5(i)–5(k)). These results were further validated with immunohistochemical staining of an independent tissue microarray (TMA) of prostate cancer patients kindly provided by University Hospital Bonn. To test for secernin-1 expression, 124 tumor free samples, 49 intraepithelial neoplasia lesions (PIN), 52 patients without biochemical relapse, and 16 patients with biochemical relapse were analyzed. The results of this TMA showed the same regulation of secernin-1 as the previous immunohistochemical analysis: tumor-free tissues showed significantly higher secernin-1 expression than tumors (*P* < 0.001), but no difference was found between patients with and without relapse (Figures 5(a)–5(h)). Immunohistochemical secernin-1 staining could detect prostate cancer with a sensitivity of 98.0% and a specificity of 99.2% for a threshold score of ≥1 versus <1. For a more precise scoring, an adapted Remmele score was used to classify the secernin-1 expression, and the results are shown in Figures 5(a)–5(h) and Supplementary Table 14. The PIN showed higher secernin-1 expression levels than the tumors but lower secernin-1 expression than the tumor-free tissues (*P* < 0.01).

*Vinculin*. Because secernin-1 is downregulated in prostate cancer, it was considered to be a potential biomarker candidate for the diagnosis of prostate cancer but was not suitable for the early detection of a recurrence.

**Figure 6:**
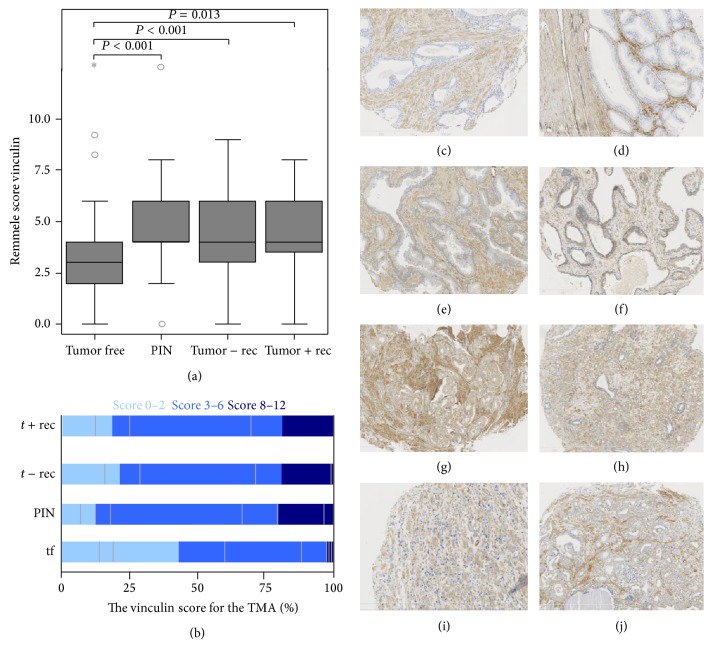
Representative immunohistochemistry of vinculin in an independent tissue microarray (TMA) obtained from the University Hospital Bonn. 116 tumor-free tissue samples, 54 prostatic intraepithelial neoplasia (PIN) lesions, 54 prostatic samples from patients without relapse (− rec), and 16 prostatectomy samples from prostate cancer patients with relapse (+ rec) were analyzed. Boxplots of the immunohistological scores of the stained tissue. (b) Percentage of each score in each analyzed patients group. For more detailed information, an adapted Remmele score was used to classify the vinculin expression. (c)–(j) Immunohistochemically stained tissue: (c) and (d) tumor-free prostatectomy samples, (e) and (f) prostatectomy samples of PIN, (g) and (h) prostatectomy samples of prostate cancer patients without relapse, and (i) and (j) prostatectomy samples of prostate cancer patients with relapse. Vinculin is significantly upregulated in peri-intraepithelial neoplasia (PIN) and prostate cancer compared to tumor-free tissue samples (*P* < 0.001).

Vinculin was validated as a potential biomarker candidate for recurrent prostate cancer. Immunohistochemical analysis of the previously described TMA set showed a significant upregulation of vinculin in PIN and prostate cancer compared to tumor-free tissue (*P* < 0.001 for tumor-free* versus* PIN and *P* < 0.001 for tumor-free* versus* prostate cancer patients without relapse and *P* = 0.013 for tumor free* versus* prostate cancer patients with relapse). Immunohistochemical vinculin staining could detect prostate cancer with a sensitivity of 38.0% and a specificity of 56.9% for a threshold score ≤1 versus >1. Biochemical prostate cancer recurrence could be detected with specificity of 65.5% and a sensitivity of 50.0%. Detailed scoring information as well as representative examples of immunohistochemical staining are shown in Figure 6 and Supplementary Table 15.

### 3.4. Validation of Potential Prostate Cancer Biomarker Candidates in Urine

**Figure 7:**
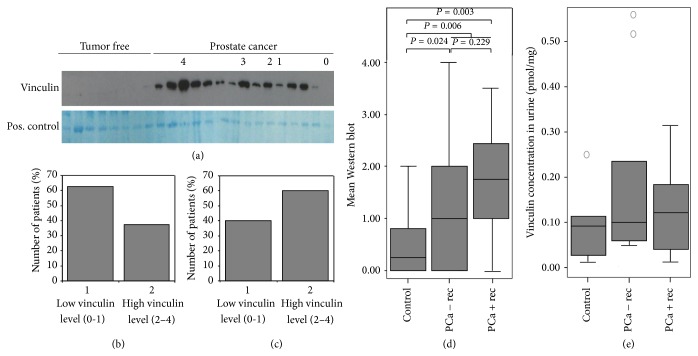
Validation attempts of vinculin levels. (a) Representative Western blot results of the vinculin levels in urine of prostate cancer patients and control patients. Coomassie brilliant blue stained gel as a positive control (pos. control). (b) Percentage of patients without recurrence with high and low vinculin levels in urine. (c) Percentage of patients with recurrence with high and low vinculin levels in urine. (d) Results of all 34 analyzed patients without recurrence, 15 prostate cancer patients with relapse and 12 analyzed control urines: boxplot of the vinculin levels in prostate cancer patients without (− rec) and with recurrence (+ rec) compared to control patients. Vinculin shows a tendency to be upregulated in prostate cancer patients with recurrence compared to patients without recurrence (*P* = 0.229). Moreover, vinculin is significantly upregulated in prostate cancer patients compared to control patients (*P* = 0,006). (e) MRM analysis of seven urine samples from control patients without prostate cancer, nine prostate cancer patients without recurrence and seven prostate cancer patients with recurrence. Vinculin (peptide SLGEISALTSK) is upregulated in prostate cancer patients urine compared to the urine of control patients (*P* = 0.438).

*Vinculin.* To validate vinculin as a potential noninvasive biomarker candidate for prostate cancer, we determined the abundance of vinculin in the urine of prostate cancer patients. Urine from 14 control patients without prostate cancer, 33 prostate cancer patients without relapse, and 15 patients with relapse were analyzed using Western blot. Vinculin expression was scored from 0 (no vinculin antibody signal detectable) to 4 (strong vinculin antibody signal detectable). Again, vinculin expression was significantly higher in the urine of prostate cancer patients than in controls (*P* = 0.006, Figure 7). Most importantly, vinculin levels in urine from prostate cancer patients with relapse were higher than in urine from patients without relapse (median score without relapse 1.00; median with relapse 1.75; *P* = 0.229; median vinculin score in the control urine 0.250; *P* = 0.006). Moreover, 62.5% of the patients without relapse showed no vinculin or low vinculin levels (score 0-1) while only 37.5% of these patients showed high vinculin levels (score 2–4). In contrast, only 40% of patients with relapse showed low vinculin levels (score 0-1) while 60% of these patients showed high vinculin levels (score 2–4). Vinculin levels in Western blots of urine >1 could detect prostate cancer with a sensitivity of 54.2% and a specificity of 85.7%. Biochemical prostate cancer recurrence could be detected with specificity of 60.6% and a sensitivity of 40.0%.

Because Western blot analysis is not practical for daily routinely use in the clinic, we tested the ability of multiple reaction monitoring (MRM) to detect vinculin abundance in urine. In the MRM-MS analysis of vinculin, 16 prostate cancer patients (nine without relapse and seven with relapse) and seven control urine samples were used. Vinculin could be detected in concentrations up to 0.55 pmol/mg protein in patients' urine. Moreover, vinculin levels were higher in prostate cancer patients' urine (median 0.109 pmol/mg) than in the urine of the control group (median 0.090 pmol/mg). Notably, the vinculin levels in urine from patients with relapse were higher (median 0.120 pmol/mg) than the vinculin levels from patients without relapse (median 0.100 pmol/mg) (Figure 7).

### 3.5. Validation of Additional Proteins as Potential Biomarker Candidates in Urine Using MRM-MS

**Figure 8:**
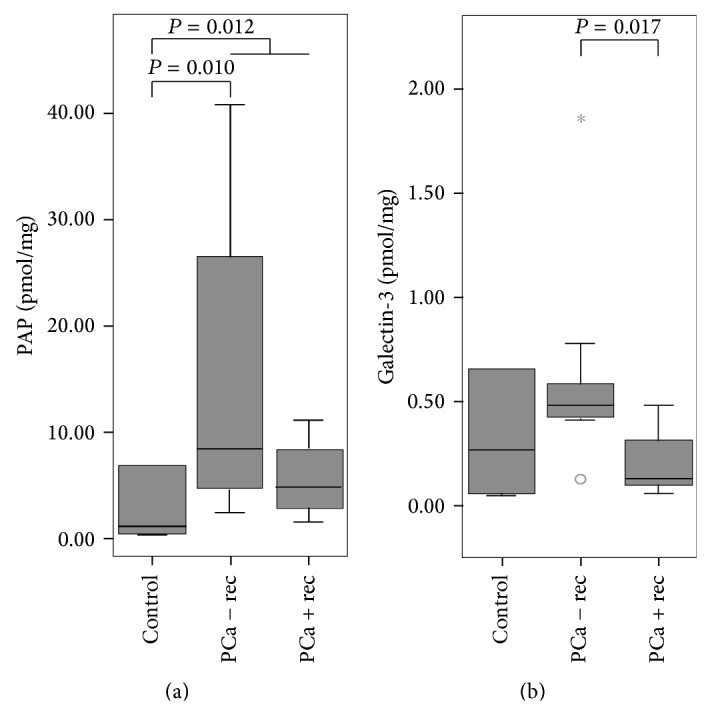
MRM analysis of prostatic acid phosphatase (PAP) peptide FQEELESETLK and galectin-3 peptide IALDFQR in nine urine samples obtained from prostate cancer (PCa) patients without relapse (− rec), seven urine samples from patients with relapse (+ rec), and seven samples from control patients. (a) PAP showed significantly higher protein levels in urine of prostate cancer patients compared to control patients urine (*P* = 0.012), while galectin-3 showed significantly lower protein levels in urine of prostate cancer patients with recurrence compared to urine of prostate cancer patients without recurrence (*P* = 0.017) (b).

*PAP, Galectin-3, and Secernin-1.* Three proteins in our initial 2D-DIGE and MS experiments were found to be associated with prostate cancer (secernin-1, PAP, and galectin-3) were chosen for a proof of principal MRM-MS study, using urine from nine prostate cancer patients without relapse and seven patients with relapse. Urine samples from seven patients without prostate cancer were used as controls. In MRM-MS results, PAP was found to show higher protein levels in the urine of prostate cancer patients compared to the PAP concentrations in the urine of the control group (median control urine = 1.21 pmol/mg median prostate-cancer patient urine = 6.26 pmol/mg; *P* = 0.012, Figure 8). However, no significant difference in PAP concentration in the urine of patients with and without relapse was found. Galectin-3 showed significantly lower protein levels in urine from prostate-cancer patients with relapse compared to urine from patients without relapse (median control urine = 0.27 pmol/mg, median in urine of prostate-cancer patients without relapse = 0.48 pmol/mg; median in urine of prostate-cancer patients with relapse = 0.13 pmol/mg; *P* = 0.017, Figure 8). Secernin-1 was not detected in the patient urine samples.

## 4. Discussion

### 4.1. Identifying Potential New Biomarker Candidates for Prostate Cancer

Ten potential biomarker candidates for prostate cancer diagnosis and 32 potential prognostic biomarker candidates to discriminate nonrecurrent from recurrent prostate cancer were identified using 2D-DIGE and MS. Ingenuity pathway analysis (IPA) was performed in order to classify the identified proteins. A comparison of tumor and tumor-free tissue revealed ten general prostate cancer biomarker candidates. These ten proteins were differentially regulated between tumor and tumor-free tissue and are mostly associated with cellular assembly, cellular development, cell morphology, cellular compromise, and carbohydrate metabolism. The 32 identified potential biomarker candidates for recurrence in prostate cancer are associated with cellular growth and proliferation, cellular development, cellular movement, cell morphology, and carbohydrate metabolism.

Potential biomarker candidates from both comparisons are associated with cellular development, cell morphology, and carbohydrate metabolism. Cellular assembly and cellular compromise are more associated with general prostate cancer biomarker candidates than with specific biomarker candidates for recurrence. In recurrent prostate cancer, proteins involved in cellular movement, especially invasion and migration, as well as cellular growth and proliferation, are often deregulated. This is in agreement with well-known features of cancer: activating invasion is crucial for the spread of cancer [57], and cellular growth and proliferation are arguably the most fundamental traits of cancer cells [57]. This study showed that cellular movement, particularly invasion and migration, cell growth, and proliferation play a more important role in recurrent prostate cancer than in prostate cancer in general.

The fact that these functions are less associated with general prostate cancer biomarker candidates than with recurrent prostate cancer might reflect the heterogeneity of the disease: as mentioned previously, many prostate cancers are indolent and are not clinically relevant due to very slow proliferation [8]. Therefore, proteins characteristic for proliferation and cellular movement are more especially suitable biomarker candidates for recurrence.

Principal component analysis (PCA) led to the detection of several potential prostate cancer biomarker candidates that have already been discussed as potential prognostic prostate cancer biomarkers in the literature. This underlines the quality of our study. PCA allowed us to detect a clear separation between benign and malignant prostate tissue as well as tissue from recurrent and nonrecurrent prostate cancer in patients. In addition, we also found differential expression levels of PAP and galectin-3, proteins which have already been discussed in literature as potential biomarker candidates for recurrent prostate cancer [58–63].

A. B. Gutman and E. B. Gutman identified increased PAP levels in patients with prostate cancer [64]. Thus, PAP was the first serum biomarker for prostate cancer to be used in clinical practice, although it lacked sufficient sensitivity to be a reliable biomarker for response to systemic therapy or recurrence [65]. Therefore, PAP was replaced by the more sensitive marker PSA. However, there is currently new interest in serum PAP as a possible prognostic marker, particularly in the prognosis of intermediate to high-risk prostate cancer [59, 60]. Fang et al. showed that, in higher-risk prostate cancer, PAP could better predict cancer-specific survival after treatment than PSA concentration and the Gleason score [58]. Surprisingly little is known about PAP as a potential biomarker for prostate cancer in urine. In 1991, Bogdanowicz et al. published a study which compared PAP and PSA levels in urine of prostate cancer patients. They found that PSA and PAP levels increased with each advancing clinical stage. PAP was elevated in 77% of patients with metastatic cancer [66]. Because we found a higher PAP abundance in recurrent prostate cancer compared to nonrecurrent prostate cancer in our initial 2D-DIGE study of prostate cancer tissue, we used MRM-MS to determine the PAP concentrations in the urine of prostate cancer patients with and without relapse. The detected concentration of PAP was significantly higher in urine of patients with recurrence compared to urine of patients without recurrence and in the control urine. Our results therefore support the hypothesis that PAP might be a good noninvasive prognostic biomarker candidate for prostate cancer. Moreover, PAP might be a noninvasive, urinary biomarker candidate for recurrent prostate cancer. As our patients' cohort was small, however, further analysis is needed to validate PAP as a promising noninvasive, urinary biomarker candidate for recurrent prostate cancer.

The 2D-DIGE experiments revealed galectin-3 as another potential new biomarker candidate for recurrent prostate cancer. Galectin-3 is similar to PAP which is already discussed as a potential biomarker candidate for recurrent prostate cancer in the literature. Galectin-3 levels have been found to be significantly decreased in primary carcinomas and metastatic disease compared to normal and premalignant prostatic tissue [61]. Normal glands show moderate galectin-3 expression in the nucleus and cytoplasm. In contrast, galectin-3 is not expressed or its expression is decreased in prostatic cancer cells and it is only present in the cytoplasm [62, 67]. Moreover, cytoplasmic expression of galectin-3 was associated with PSA relapse in univariate analysis [62]. Multivariate analysis revealed galectin-3 as an independent predictor for PSA relapse after the Gleason score and pathological stage [62]. Thus, galectin-3 promotes tumor progression when expressed in the cytoplasm but shows antitumor activity when expressed in the nucleus [62, 68]. Additionally hormone-refractory tumors showed lower galectin-3 expression than hormone-sensitive tumors [63]. Interestingly, galectin-3 is downregulated in the serum of prostate cancer patients compared to serum of healthy controls [69].

To the best of our knowledge, galectin-3 levels in urine of prostate cancer patients have not yet been studied. The only published data for galectin-3 in urine is a combined assay of galectin-3 methylation specific PCR in combination with GSTP1 (Glutathione S-transferase P1) promoter methylation. In that study, galectin-3 promoter methylation had 100% specificity and could be detected in 22/22 urine samples of patients [70]. In our study, we analyzed galectin-3 levels in urine of prostate cancer patients using MRM. Consistent with our 2D-DIGE results and other published data, we were able to detect galectin-3 downregulation in the urine of prostate cancer patients with recurrence compared to patients without recurrence. Thus, galectin-3 levels in urine are a promising biomarker candidate for the prediction of prostate cancer progression.

2D-DIGE analysis also revealed lower secernin-1 abundance in recurrent prostate cancer compared to nonrecurrent prostate cancer. Validation of secernin-1 expression using tissue Western blots, immunohistochemical staining, and immunohistochemical staining of an independent TMA set showed a significant downregulation of secernin-1 in prostate cancer tissue compared to tumor-free tissue. The downregulation of secernin-1 already starts at the point of PIN. Secernin-1 is described as being deregulated in different carcinomas: secernin-1 is upregulated in colon cancer [71–73], in gastric cancer [73], and in Barrett's esophagus associated with high-grade dysplasia compared to nondysplastic Barrett's esophagus [74]. Moreover, the upregulation of secernin-1 in colorectal cancer compared to normal colorectal tissue correlates with clinicopathological data and is associated with poor prognosis [72]. In contrast to these results, Suehara et al. described secernin-1 as being upregulated in synovial sarcoma with good prognosis compared to synovial sarcoma with poor prognosis [75]. In prostate cancer, Ashida et al. found KIAA0193 (secernin-1) to be downregulated during the transition from PIN to prostate cancer using genome-wide gene expression profiling [6]. In summary, secernin-1 is up regulated in many tumors, but we and others have demonstrated that secernin-1 is downregulated in prostate cancer tissue compared to tumor-free prostatic tissue. Thus, secernin-1 is a novel potential biomarker candidate for recurrent prostate cancer.

Our immunohistochemical analysis revealed secernin-1 expression in the basal layer, while prostatic luminal cells showed no secernin-1 expression. Notably, some of the tumor glands of the immunohistochemically analyzed tumor sample set in the explorative phase showed a weak secernin-1 expression (data not shown). This secernin-1 expression could not be confirmed in the independent TMA obtained from the University Hospital Bonn. Thus, it appears that secernin-1 is mostly expressed in the basal layer and could possibly be lost during the transition from benign prostate tissue to PIN and prostate cancer. As secernin-1 expression is identical in inflamed and normal prostate tissue, secernin-1 might be a good biomarker candidate for prostate cancer. Secernin-1 level in urine has not been detected by MRM-MS thus far, so the potential of secernin-1 as a noninvasively obtained biomarker candidate in urine from prostate cancer patients is still part of our ongoing work.

Our 2D-DIGE analysis showed that vinculin abundance is higher in recurrent prostate cancer compared to nonrecurrent prostate cancer tissue. Ruiz et al. showed that vinculin is not expressed or only expressed at a very low level in benign prostate hyperplasia. In 92% of the localized prostate cancer samples, vinculin showed a low expression level. Vinculin showed high expression levels in 16% of castration-resistant prostate cancer samples and a moderate expression level in 34% of castration resistant prostate cancer samples [76]. Our TMA analysis also showed that vinculin is significantly upregulated in prostate cancer tissue compared to tumor-free prostatic tissue. This upregulation can already be found in PIN lesions. The Western blot analysis of urine confirmed the significant upregulation of vinculin in prostate cancer patients. Moreover, vinculin showed higher protein levels in the urine of prostate cancer patients with recurrence compared to the urine from patients without recurrence. Thus, future analysis of a larger number of patient samples may validate the potential of vinculin as a biomarker candidate for recurrent prostate cancer. Pang et al. found that vinculin isoform 2 was downregulated in the lymph nodes of metastatic cancer patients compared to lymph node-negative cancer tissues [33]. Thorsen et al. described an alternative splicing variant of vinculin in colon, bladder, and prostate cancer where exon 19 of the vinculin gene is skipped in the cancer tissue, and the long isoform was downregulated in metastatic prostate cancer compared to localized cancer [77]. These two studies showed that different vinculin isoforms may be differentially expressed in prostate cancer. Thus vinculin expression and vinculin isoforms in prostate cancer should be analyzed carefully in the future.

## 5. Conclusion

2D-DIGE combined with MS is a labor-intensive and expensive method with a low throughput of the 2D-DIGE. But the advantages of the proteomic methods compared to DNA methods are magnificent: most functional information about cancer-related genes are placed in the proteome [78] and the discordance of mRNA and protein level elucidates the advantage of proteomic methods [79, 80]. Moreover, most of FDA-approved diagnostic and prognostic tests for cancer (therapies) are protein-based, which makes it easier to transfer results of protein studies to clinical useful tools than results of genome-based studies [81]. 2D-DIGE enables the separation of thousands of proteins [82], including protein isoforms [32] and posttranslational modifications [83]. Moreover, it has a linear detection rate of >10.000 of protein abundances [39, 82]. Unfortunately, very big or very small proteins, plasma membrane-associated proteins, hydrophobic, very alkaline, or very acidic protein scan not be detected using 2D-DIGE [84]. Moreover, gel-to-gel variability is around 20%. Therefore, 3–5 biological replicates per analyzed group are needed to detect expression differences of a protein from 1.5 to 2 [85]. These disadvantages can be minimized by separating one sample and a standard in each gel [86]. Label-free proteomic approaches are an alternative to 2D-DIGE for analyzing the proteome. But Megger et al. could show that different proteins can be detected by 2D-DIGE and label-free methods. Therefore, both methods complement to each other [53].

In conclusion, we have successfully demonstrated that 2D-DIGE combined with MS is a powerful tool to identify differentially expressed proteins in prostate cancer tissue. Galectin-3 was verified as a potential prognostic biomarker candidate by analyzing prostate cancer patient urine samples with MRM-MS. MRM-MS was also found to be suitable for quantifying PAP levels in urine. Secernin-1 has been successfully validated as a potential diagnostic biomarker candidate for prostate cancer in tissue using Western blot analysis. Vinculin expression has been shown to be upregulated in prostate cancer patient tissue samples, while the urine of recurrent prostate cancer patients showed higher vinculin levels than nonrecurrent prostate cancer patients. Although larger-scale validation studies involving more patients are still needed, vinculin and galectin-3 seem to be promising urinary biomarker candidates for recurrent prostate cancer, while secernin-1 seems to be a useful tissue diagnostic biomarker candidate for prostate cancer.

## Supplementary Material

Identification and Validation of Potential New Biomarkers for Prostate Cancer Diagnosis and Prognosis Using 2D-DIGE and MS” by Cordelia Geisler, Nadine T. Gaisa, David Pfister, Susanne Fuessel, Glen Kristiansen, Till Braunschweig, Sonja Gostek, Birte Beine, Hanna C. Diehl, Angela Jackson, Christoph H. Borchers, Axel Heidenreich, Helmut E. Meyer, Ruth Knüchel and Corinna Henkel. Supplementary tables 1-7 give detailed information about the used patient samples while supplementary tables 8 and 9 give detailed information about the SIS peptides used for MRM. Supplementary table 9 and 10 list details about the 2D-DIGE results while supplementary table 11 and 12 give details about the MS identification results. Supplementary table 14, 15 and supplementary figure 1 give detailed information about the immunohistochemical analysis.

## Figures and Tables

**Table 1:** Sample sets used in the experiments.

Experiment	Sample set			
	Frozen tissue (obtained from University Hospitals Dresden and Aachen)	FFPE tissue (obtained from University Hospital Dresden)	TMA (obtained from University Hospital Bonn)	Urine samples (obtained from University Hospital Aachen)
IDENTIFICATON				
2D-DIGE and MS-analysis	X			
VALIDATION (TISSUE)				
Western blot secernin-1	X			
IH secernin-1		X		
TMA secernin-1			X	
TMA vinculin			X	
VALIDATION (URINE)				
Western blot vinculin				X
MRM vinculin				X
MRM PAP				X
MRM galectin-3				X

**Table 2:** Antibodies used for Western blot analysis.

Antibody	Host	Type	Company	Dilution
*β*-Actin (A5441)	Mouse	Monoclonal	Sigma Aldrich, St. Louis, USA	1 : 500
Secernin-1	Rabbit	Polyclonal	Sigma Aldrich, St. Louis, USA	1 : 500
Vinculin	Mouse	Monoclonal	Fitzgerald, North Acton, USA	1 : 1,000
Anti-mouse + HRP (P0447)	Goat	Polyclonal	DAKO, Hamburg, Germany	1 : 5,000
Anti-rabbit + HRP (P0448)	Goat	Polyclonal	DAKO, Hamburg, Germany	1 : 5,000
Peroxidase anti-Mouse IgG (PI-2000)	Horse	Polyclonal	Vector Laboratories, USA	1 : 10,000
Peroxidase anti-Rabbit IgG (PI-1000)	Goat	Polyclonal	Vector Laboratories, USA	1 : 20,000

**Table 3:** Antibodies used for immunohistochemistry.

Target protein	Species	Type	Company	Dilution	Incubation time and temperature	Positive control
Secernin-1	Rabbit	Polyclonal	Sigma Aldrich, St. Louis, USA	1 : 1000	1 h, 37°C	Testis
Vinculin	Mouse	Monoclonal	Fitzgerald, North Acton, USA	1 : 1000	Overnight, 4°C	Testis

**Table 4:** Deregulated proteins in prostate cancer identified with 2D-DIGE and MS.

Spot		Acc. no.	Protein name	Ratio Tf versus Tu	*P* value (*t*-test) Tf versus Tu	*P* value (*U*-test) Tf versus Tu
Down 01		P22626	Heterogeneous nuclear ribonucleoproteins A2/B1	−21.8	0.043	0.035
Down 05		P17661	Desmin	−15.7	0.156	0.024
Down 06		P17661	Desmin	−9.1	0.085	0.009
Down 04		P17661	Desmin	−3.8	0.037	0.012
Down 09		P09493	Tropomyosin alpha-1 chain	−2.9	0.035	0.042
Down 10		P09493	Tropomyosin alpha-1 chain	−2.9	0.009	0.020
Down 08		P09493	Tropomyosin alpha-1 chain	−2.8	0.010	0.019
Down 07		P12277	Creatine kinase B-type	−2.7	0.045	0.009
Down 11		Q05682	Caldesmon	−2.2	0.115	0.026
Down 02		P08670	Vimentin	−1.7	0.053	0.024
Down 03		P17661	Desmin	−1.6	0.039	0.110
Up 01		COEA1	Collagen alpha-1(XIV) chain	2.4	0.104	0.042
Up 03	Mix	ANXA5	Annexin A5	3.7	0.038	0.033
		A1BG	Alpha-1B-glycoprotein	3.7	0.038	0.033
		P04217	Alpha-1B-glycoprotein	3.7	0.038	0.033
Up 02		TCPA	T-complex protein 1 subunit alpha	46.7	0.405	0.049

2D-DIGE: two-dimensional differences in gel electrophoresis; MS: mass spectrometry; Acc. no.: accession number; Tf: tumor free; Tu: tumor; *U*-test: two-sided Mann-Whitney *U*-test; ratio: division of the mean; mean: normalized spot volume.

**Table 5:** Deregulated proteins in recurrent prostate cancer identified with 2D-DIGE and MS.

Spot		Acc. no.	Protein name	Ratio + versus − rec	*P* value (*t*-test) + versus − rec	*P* value (*U*-test) + versus − rec
Down 13		P01857	Ig gamma-1 chain C region	**−274.3**	0.334	0.041
Down 01		FLNA	Filamin-A	**−12.0**	0.269	0.031
Down 12		SCRN1	Secernin-1	**−8.9**	0.088	0.044
Down 04		O95394	Phosphoacetylglucosamine mutase	**−7.5**	0.228	0.012
Down 08		PYGB	Glycogen phosphorylase, brain form	**−5.6**	0.176	0.031
Down 03		P06396	Gelsolin	**−5.3**	0.092	0.023
Down 02		P06396	Gelsolin	**−3.5**	0.109	0.019
Down 16		LEG3	Galectin-3	**−3.4**	0.272	0.033
Down 05		PTGR2	Prostaglandin reductase 2	**−3.2**	0.047	1.169
Down 06		LMNA	Lamin-A/C	**−2.6**	0.036	0.023
Down 14		TTC38	Tetratricopeptide repeat protein 38	**−2.4**	0.087	0.036
Down 15	Mix	MDHM	Malate dehydrogenase, mitochondrial	**−2.3**	0.233	0.014
		P51911	Calponin-1	**−2.3**	0.233	0.014
Down 09		P16870	Carboxypeptidase E precursor	**−2.2**	0.122	0.049
Down 10		CO6A2	Collagen alpha-2(VI) chain	**−1.9**	0.018	0.031
Down 07		LMNA	Lamin-A/C	**−1.7**	0.133	0.036
Down 11		G6PD	Glucose-6-phosphate 1-dehydrogenase	**−1.7**	0.255	0.036
Up 08	Mix	Q9UBR2	Cathepsin Z precursor	**1.2**	0.529	0.951
		P20774	Mimecan precursor	**1.2**	0.529	0.951
Up 10		CAZA1	F-actin-capping protein subunit alpha-1	**1.5**	0.209	0.042
Up 05		PAPP	Prostatic acid phosphatase	**1.8**	0.048	0.056
Up 01		VINC	Vinculin	**2.2**	0.027	0.031
Up 03		GRP78	78 kDa glucose-regulated protein	**2.5**	0.025	0.036
Up 02		COEA1	Collagen alpha-1(XIV) chain	**2.5**	0.075	0.049
Up 09		KCD12	BTB/POZ domain-containing protein KCTD12	**2.6**	0.158	0.045
Up 11		GLO2	Hydroxyacylglutathione hydrolase, mitochondrial	**3.2**	0.047	0.042
Up 12	Mix	ANXA5	Annexin A5	**3.2**	0.032	0.074
		A1BG	Alpha-1B-glycoprotein	**3.2**	0.032	0.074
Up 04		SYNEM	Synemin	**4.9**	0.055	0.027
Up 13		PRDX4	Peroxiredoxin-4	**4.9**	0.022	0.004
Up 06		ANXA4	Annexin A4	**6.4**	0.288	0.042
Up 07		TCPA	T-complex protein 1 subunit alpha	**51.9**	0.275	0.031

2D-DIGE: two-dimensional differences in gel electrophoresis; MS: mass spectrometry; Acc. no.: accession number; + rec: prostate cancer with recurrence; − rec: prostate cancer without recurrence; *U*-Test: two-sided Mann-Whitney *U*-Test; ratio: division of the mean; mean: normalized spot volume.

**Table 6:** Ingenuity pathway analysis of the deregulated proteins in prostate cancer, identified by 2D-DIGE and MS localization; type and top 5 functions of the identified proteins are listed.

ID	Entrez gene name	Localization	Type	Functions (top 5)
P04217	Alpha-1-B glycoprotein	Extracellular space	Other	
P08758	Annexin A5	Plasma membrane	Other	Cellular assembling and organization, carbohydrate metabolism, and cellular compromise
Q05682	Caldesmon 1	Cytoplasm	Other	Cellular assembling and organization, cell morphology, and development of connective tissue
P12277	Creatine kinase, brain	Cytoplasm	Kinase	
Q05707	Collagen, type XIV, alpha 1	Extracellular space	Other	Cellular development and development of connective tissue
P17661	Desmin	Cytoplasm	Other	Cellular assembling and organization, development and function of the cardiovascular system, and organ morphology
P22626	Heterogeneous nuclear ribonucleoprotein A2/B1	Nucleus	Other	Cellular development
P17987	T-complex 1	Cytoplasm	Other	
P09493	Tropomyosin 1 (alpha)	Cytoplasm	Other	Cellular assembling and organization, cell morphology, cellular development, development of connective tissue, development and function of the cardiovascular system, and organ morphology
P08670	Vimentin	Cytoplasm	Other	Cellular assembling and organization, cell morphology, cellular development, development of connective tissue, development and function of the cardiovascular system, and tumor morphology
P04217	Alpha-1-B glycoprotein	Extracellular space	Other	
P15309	Acid phosphatase, prostate	Extracellular space	Phosphates	Cellular growth and differentiation, cellular development, and development and function of the hematopoietic system
P09525	Annexin A4	Plasma membrane	Other	
P08758	Annexin A5	Plasma membrane	Other	Carbohydrate metabolism, organ development, and development and function of the hematopoietic system
P52907	Capping protein (actin filament) muscle Z-line, alpha 1	Cytoplasm	Other	Cellular growth and differentiation
P51911	Calponin 1, basic, smooth muscle	Cytoplasm	Other	Cellular growth and differentiation and cellular development
Q05707	Collagen, type XIV, alpha 1	Extracellular space	Other	Cellular growth and differentiation, cellular development, and development and function of connective tissues
P12110	Collagen, type VI, alpha 2	Extracellular space	Other	Cellular growth and differentiation
P16870	Carboxypeptidase E	Cytoplasm	Peptidase	
Q9UBR2	Cathepsin Z	Cytoplasm	Peptidase	Cellular growth and differentiation, cellular development, cell morphology, cellular movement (migration), and development and function of the hematopoietic system
P21333	Filamin A, alpha	Cytoplasm	Other	Cell morphology, cellular movement (migration), and development and function of the cardiovascular system
P11413	Glucose-6-phosphate dehydrogenase	Cytoplasm	Enzyme	Cellular development, carbohydrate metabolism, organ development, and development and function of the hematopoietic system
P06396	Gelsolin	Extracellular space	Other	Cellular growth and differentiation, cellular movement (invasion), organ development, and development and function of connective tissue
Q16775	Hydroxyacylglutathione hydrolase	Cytoplasm	Enzyme	Organ development
P11021	Heat shock 70 kDa protein 5 (glucose-regulated protein, 78 kDa)	Cytoplasm	Enzyme	Cellular growth and differentiation, cellular development, cellular movement (migration), development and function of the hematopoietic system, and development and function of the cardiovascular system
P01857	Immunoglobulin heavy constant gamma 1 (G1m marker)	Extracellular space	Other	Cellular growth and differentiation, cellular development, cellular movement (migration, invasion), development and function of the hematopoietic system, and development and function of the cardiovascular system
Q96CX2	Potassium channel tetramerization domain containing 12	Plasma membrane	Ion channel	
P17931	Lectin, galactoside-binding, soluble, 3	Extracellular space	Other	Cellular growth and differentiation, cellular development, cell morphology, cellular movement (migration, invasion, and chemotaxis), organ development, development and function of the hematopoietic system, development and function of connective tissue, and development and function of the cardiovascular system
P02545	Lamin A/C	Nucleus	Other	Cell morphology
P40926	Malate dehydrogenase 2, NAD (mitochondrial)	Cytoplasm	Enzyme	
P20774	Osteoglycin	Extracellular space	Growth factor	
O95394	Phosphoglucomutase 3	Cytoplasm	Enzyme	
Q13162	Peroxiredoxin 4	Cytoplasm	Enzyme	Cellular growth and differentiation, cellular development, and development and function of connective tissues
Q8N8N7	Prostaglandin reductase 2	Cytoplasm	Enzyme	
P11216	Phosphorylase, glycogen; brain	Cytoplasm	Enzyme	
Q12765	Secernin 1	Cytoplasm	Other	
O15061	Synemin, intermediate filament protein	Cytoplasm	Other	Cellular growth and differentiation, cellular development, cell morphology, and cellular movement (migration)
P17987	T-complex 1	Cytoplasm	Other	
Q5R3I4	Tetratricopeptide repeat domain 38	unknown	Other	
P18206	Vinculin	Plasma membrane	Enzyme	Cellular movement (migration) and development and function of connective tissues
