# Supplementary material for: Identification and Validation of Potential New Biomarkers for Prostate Cancer Diagnosis and Prognosis Using 2D-DIGE and MS
Source: Biomed Res Int. 2015 Jan 15;2015:454256. doi: 10.1155/2015/454256 (PMC4312578; doi:10.1155/2015/454256)
Supplement: Supplementary file 1 — Identification and Validation of Potential New Biomarkers for Prostate Cancer Diagnosis and Prognosis Using 2D-DIGE and MS” by Cordelia Geisler, Nadine T. Gaisa, David Pfister, Susanne Fuessel, Glen Kristiansen, Till Braunschweig, Sonja Gostek, Birte Beine, Hanna C. Diehl, Angela Jackson, Christoph H. Borchers, Axel Heidenreich, Helmut E. Meyer, Ruth Knüchel and Corinna Henkel. Supplementary tables 1-7 give detailed information about the used patient samples while supplementary tables 8 and 9 give detailed information about the SIS peptides used for MRM. Supplementary table 9 and 10 list details about the 2D-DIGE results while supplementary table 11 and 12 give details about the MS identification results. Supplementary table 14, 15 and supplementary figure 1 give detailed information about the immunohistochemical analysis. [file 454256.f1.pdf]

Supplementary Data

Supplementary table 1: Clinicopathological characteristics of prostate cancer patients tissue used for 2D-DIGE analysis

Variable	Categorization	Patients without recurrence		Patients with recurrence	
		n[a]	%	n[a]	%
Mean age [years]		60.9 (47 – 75)		62.56 (54 – 70)	
	<62 years	7	58.3	4	36.4
	≥62 years	5	41.7	5	45.5
	no information	0	0	2	18.2
Tumor size					
	pT2	6	50.0	5	45.5
	pT3	6	50.0	4	36.4
	pX	0	0	2	18.2
Lymph node status					
	pN0	10	83.3	9	81.8
	pN1	1	8.3	0	0
	pNx	1	8.3	2	18.2
Resection margin					
	R0	6	50.0	8	72.7
	R1	2	16.7	0	0
	Rx	4	33.3	3	27.3
Gleason Score					
Mean Gleason Score		7.6 (7 – 9)		7.1 (6 – 8)	
	6	0	0	1	9.1
	7	7	58.3	6	54.5
	8	2	16.7	2	18.2
	9	3	25.0	0	0
	no information	0	0	2	18.2
PSA					
Mean PSA [ng/ml]		16.79 (5.37 – 28.46)		9.98 (1.29 – 22.50)	
	0 – 4 ng/ml	0	0	1	9.1
	4-10 ng/ml	3	25.0	4	36.4
	>10 ng/ml	6	50.0	4	36.4
	no information	3	25.0	2	18.2
Follow up					
Mean follow-up time [month]		69.4 (35 – 106)		54.2 (9 – 86)	
	0-24 month	0	0	1	9.1
	24-60 month	5	41.7	7	63.6
	> 60 month	7	58.3	3	27.3
	no information	0	0	0	0

Fourteen tumor-free tissue samples corresponding to the tumor samples were used as tumor free controls. The average age of the selected patients was 61.0 years (55 – 69 years), excluding the age of four patients as these were unknown. The mean concentration of the initial PSA was 13.24 ng/ml (3.39 – 22.50 ng/ml), excluding the initial PSA of five patients as these were unknown.

Supplementary table 2: Clinicopathological characteristics of prostate cancer patients tissue used for western blot analysis of secernin-1

Variable	Categorization	Patients without recurrence		Patients with recurrence	
		n[a]	%	n[a]	%
Mean age [years]		64.5 (56-75)		62.83 (58 – 67)	
	<63 years	2	50	2	33.3
	≥63 years	2	50	4	66.7
Tumor size					
	pT2	1	25	4	66.7
	pT3	3	75	2	33.3
Lymph node status					
	pN0	4	100	5	83.3
	pN1	0	0	1	16.7
Resection margin					
	R0	4	100	6	100.0
Gleason Score					
Mean Gleason Score		7.33 (7 – 9)		8.25 (7 – 9)	
	7	1	25	5	83.3
	8	1	25	0	0
	9	2	50	1	16.7
PSA					
Mean PSA [ng/ml]		7.03 (4.32 – 9.76)		11.59 (1.29 – 22.50)	
	4-10 ng/ml	3	75	2	33.3
	>10 ng/ml	0	0	4	66.7
	no information	1	25	0	0
Follow up					
Mean follow-up time [month]		74.5 (66 -85)		53.16 (6-75)	
	0-24 month	0	0	1	16.7
	24-60 month	0	0	2	33.3
	> 60 month	4	100	3	50.0

Tumor-free tissue areas of prostatectomies from eight prostate cancer patients were used as controls. The average age of the selected patients was 68.05 years (63 – 75 years). The mean concentration of the initial PSA was 5.41 ng/ml (0.51 – 12.70 ng/ml).

Supplementary table 3: Clinicopathological characteristics of prostate cancer patients tissue used for immunhistochemistry of secernin-1

Variable	Categorization	Patients without recurrence		Patients with recurrence	
		n[a]	%	n[a]	%
Mean age [years]		62. 9 (54.7 – 75.5)		62.8 (54.4 – 71.5)	
	<63 years	7	46.2	7	50.0
	≥63 years	6	53.8	7	50.0
Tumor size					
	pT2	5	38.5	5	35.7
	pT3	8	61.5	8	57.1
	pX	0	0	1	7.1
Lymph node status					
	pN0	11	84.6	10	71.4
	pN1	2	15.4	4	28.6
Resection margin					
	R0	9	69.2	12	58.7
	R1	2	15.4	2	14.3
	Rx	2	15.4	0	0
Gleason Score					
Mean Gleason Score		7.8 (7 – 9)		7.7 (6 – 9)	
	6	0	0	1	7.1
	7	7	46.2	6	42.9
	8	2	15.4	3	21.4
	9	4	30.8	4	28.6
PSA					
Mean PSA [ng/ml]		15.0 (4.32 – 28.64)		10.2 (1.29 – 26.07)	
	< 4 ng/ml	0	0	3	28.6
	4-10 ng/ml	5	38.5	5	35.7
	>10 ng/ml	7	46.2	6	42.9
	no information	1	7.7	0	0
Follow up					
Mean follow-up time [month]		72.2 (35 – 106)		52.2 (6 – 86)	
	0-24 month	0	0	2	14.3
	24-60 month	3	23.1	8	57.1
	> 60 month	10	76.9	4	28.6

Twenty-seven tumor-free tissue samples corresponding to the tumor samples were used as tumor free controls. Additionally, 15 prostatectomy samples of patients with benign prostate hyperplasia were analyzed. The average age of the selected patients was 69.7 years. The mean concentration of the initial PSA was 4.45 ng/ml. Clinical data from one patient was not available.

Supplementary table 4: Clinicopathological characteristics of prostate cancer patients tissue used for TMA for immunohistochemical staining of secernin-1

Variable	Categorization	Patients without recurrence		Patients with recurrence	
		n[a]	%	n[a]	%
Mean age [years]		64.8 (47 – 74)		64.9 (51 – 70)	
	<65 years	21	40.4	6	37.5
	≥65 years	31	59.6	10	62.5
Tumor size					
	pT2	36	69.2	6	37.5
	pT3	15	28.8	10	62.5
	pT4	1	1.9	0	0
Lymph node status					
	pN0	48	92.3	15	93.8
	pN1	4	7.7	1	6.3
Resection margin					
	R0	30	57.7	7	43.8
	R1	21	40.4	8	50.0
	Rx	1	1.9	0	0
Gleason Score					
Mean Gleason Score		6.10 (3 – 9)		7.56 (6 – 9)	
	3	1	1.9	0	0
	4	1	1.9	0	0
	5	15	28.8	0	0
	6	17	32.7	2	12.5
	7	12	23.1	6	37.5
	8	5	9.6	5	31.3
	9	1	1.9	3	18.8
PSA					
Mean PSA [ng/ml]		11.10 (1.02 – 163.0)		10.88 (1.51 – 50.7)	
	< 4 ng/ml	7	13.5	0	0
	4-10 ng/ml	32	61.5	4	25.0
	>10 ng/ml	11	21.2	12	75.0
	no information	2	3.8	0	0
Follow up					
Mean follow-up time [month]		66.2 (32 – 90)		10.13 (42 – 88)	
	24-60 month	19	36.5	0	0
	> 60 month	33	63.5	4	25.0
	no information	0	0	12	75.0

124 tumor-free tissue samples corresponding to the tumor samples were used as tumor free controls. The average age of the selected patients was 64.7 years (45 – 78 years). The mean concentration of the initial PSA was 13.67 ng/ml (0.98 – 395 ng/ml). Clinical data from six patients was not available.

For analyzing secernin-1 expression in intraepithelial neoplasia (PIN) 49 samples were used. The average age of these patients was 64.3 years (45 – 75 years). Clinical data from three patients was not available for this analysis.

Supplementary table 5: Clinicopathological characteristics of prostate cancer patient tissue used for the TMA for immunohistochemical staining of vinculin

Variable	Categorization	Patients without recurrence		Patients with recurrence	
		n[a]	%	n[a]	%
Mean age [years]		65.0 (47 – 74)		64.65 (51 – 71)	
	<65 years	23	41.8	6	37.5
	≥65 years	32	58.2	10	62.5
Tumor size					
	pT2	39	70.9	7	43.8
	pT3	15	27.3	9	56.3
	pT4	1	1.8	0	0
Lymph node status					
	pN0	52	94.5	15	93.8
	pN1	3	5.5	1	6.3
Resection margin					
	R0	33	60.0	8	50.0
	R1	21	38.2	7	43.8
	Rx	1	1.8	1	6.3
Gleason Score					
Mean Gleason Score		6.09 (4 – 9)		7.25 (4 – 9)	
	4	4	7.3	1	6.3
	5	15	27.3	0	0
	6	18	32.7	2	12.6
	7	14	25.5	5	31.3
	8	3	5.5	5	31.3
	9	1	1.8	3	18.8
PSA					
Mean PSA [ng/ml]		8.09 (0.98 – 35.4)		10.39 (1.51 – 50.7)	
	< 4 ng/ml	8	14.5	3	18.8
	4-10 ng/ml	32	58.2	8	50.0
	>10 ng/ml	13	23.6	4	25.0
	no information	2	3.6	1	6.3
Follow up					
Mean follow-up time [month]		65.78 (12 – 90)		67.15 (25 – 88)	
	0 – 24 month	1	1.8	0	0
	24-60 month	19	34.5	5	31.3
	> 60 month	35	63.6	11	68.8

116 tumor-free tissue samples corresponding to the tumor samples were used as tumor free controls. The average age of the selected patients was 64.7 years (45 – 76 years). The mean concentration of the initial PSA was 12.62 ng/ml (0.98 – 395 ng/ml). Clinical data from six patients was not available.

For analyzing vinculin expression in intraepithelial neoplasia (PIN), 54 samples were used. The average age of these patients was 64.4 years (47 – 75 years). Mean concentration of the initial PSA of these patients was 16.76 ng/ml (1.002 – 395 ng/ml). Clinical data from three patients was not available.

Supplementary table 6: Clinicopathological characteristics of prostate cancer patient urine used for the vinculin western blot analysis analysis

Variable	Categorization	Patients without recurrence		Patients with recurrence	
		n[a]	%	n[a]	%
Mean age [years]		63.4 (42 – 77)		63.1 (50 – 73)	
	<63 years	11	32.4	4	26.7
	≥63 years	22	64.7	11	73.3
	no information	1	2.9	0	0
Tumor size					
	pT2	23	67.6	6	40.0
	pT3	9	26.5	7	46.7
	pX	2	5.9	2	13.3
Lymph node status					
	pN0	13	38.2	5	33.3
	pNx	21	61.8	10	66.7
Resection margin					
	R0	24	70.6	7	46.7
	R1	6	17.6	6	40.0
	Rx	4	11.8	2	13.3
Gleason Score					
Mean Gleason Score		6.8 (5 – 8)		7.3 (6 – 9)	
	5	1	2.9	0	0
	6	9	26.5	2	13.3
	7	19	55.9	8	53.3
	8	3	8.8	0	0
	9	0	0	3	20.0
	no information	2	5.9	2	13.3
PSA					
Mean PSA [ng/ml]		7.25 (1.14 – 17.00)		14.99 (6.57 – 23.40)	
	0 – 4 ng/ml	2	5.9	0	0
	4-10 ng/ml	13	38.2	1	6.7
	>10 ng/ml	2	5.9	1	6.7
	no information	17	50.0	13	86.7
Follow up					
Mean follow-up time [month]		39.6 (1 – 84)		51.47 (19 – 74)	
	0-24 month	4	11.8	1	6.7
	24-60 month	25	73.5	7	46.7
	> 60 month	4	11.8	7	46.7
	no information	1	2.9	0	0

Fourteen urine samples from a healthy population over 40 years were used as controls.

Supplementary table 7: Clinicopathological characteristics of prostate cancer patient urine obtained from the University Hospital Aachen and used for MRM

Variable	Categorization	Patients without recurrence		Patients with recurrence	
		n[a]	%	n[a]	%
Mean age [years]		66.8 (60-71)		62.5 (50 -70)	
	<66 years	4	44.4	3	42.3
	≥66 years	5	55.5	4	57.1
Tumor size					
	pT2	3	33.3	3	42.3
	pT3	5	55.5	4	57.1
	pX	1	11.1	0	0
Lymph node status					
	pN0	4	44.4	3	42.3
	pNx	5	55.5	4	57.1
Resection margin					
	R0	5	55.5	3	42.3
	R1	2	22.2	4	57.1
	Rx	2	22.2	0	0
Gleason Score					
Mean Gleason Score		6.6 (5 – 8)		6.9 (6 – 7)	
	5	1	11.1	0	0
	6	3	33.3	1	14.3
	7	4	44.4	6	85.7
	8	1	11.1	0	0
PSA					
Mean PSA [ng/ml]		17.5		6.6 (6.6 – 6.6)	
	4-10 ng/ml	1	11.1	1	14.3
	>10 ng/ml	3	33.3	0	0
	no information	5	55.6	6	85.7
Follow up					
Mean follow-up time [month]		54.8 (35 -84)		49.1 (29-74)	
	24-60 month	5	55.6	5	71.4
	> 60 month	3	33.3	2	28.6
	no information	1	11.1	0	0

Urine from four patients with bladder cancer and three patients without cancer were used as controls. The average age of selected patients was 67.8 years.

Supplementary table 8: Qualifier and Quantifier transitions used for MRM analysis of prostatic acid phosphatase, galectin-3, and vinculin

		Quantifier				Qualifier 1				Qualifier 2			
**Protein / Peptide**	**Peptide** (heavy / light)	**Ion**	**Precursor**	**Product**	**CE [V]**	**Ion**	**Precursor**	**Product**	**CE [V]**	**Ion**	**Precursor**	**Product**	**CE [V]**
Galectin-3 IALDFQR	L	2y5	431.7	678.4	9	2y6	431.7	749.4	13	2y4	431.7	565.3	9
Galectin-3 IALDFQR	H	2y5	436.7	688.4	9	2y6	436.7	759.4	13	2y4	436.7	575.3	9
Galectin-3 IQVLVEPDHFK	L	3y9 ++	442.2	542.3	9	2b2	662.9	242.2	21	3b2	442.2	242.2	9
Galectin-3 IQVLVEPDHFK	H	3y9 ++	444.9	546.3	9	2b2	666.9	242.1	21	3b2	444.9	242.1	9
Galectin-3 VAVNDAHLLQYNHR	L	4y11 ++	413.2	690.8	9	3y13 ++	550.6	775.9	13	3y12 ++	550.6	740.4	13
Galectin-3 VAVNDAHLLQYNHR	H	4y11 ++	415.7	695.8	9	3y13 ++	554.0	780.9	13	3y12 ++	554.0	745.4	13
Galectin-3 GNDVAFHFNPR	L	3y7 ++	425.2	444.7	9	3y9 ++	425.2	551.8	9	3y5 ++	425.2	335.7	9
Galectin-3 GNDVAFHFNPR	H	3y7 ++	428.5	449.7	9	3y9 ++	428.5	556.8	9	3y5 ++	428.5	340.7	9
Prostatasepzifische saure Phosphatase FQELESETLK	L	2y8	612.3	948.5	17	2b2	612.3	276.1	13	2y7	612.3	819.5	21
Prostatasepzifische saure Phosphatase FQELESETLK	H	2y8	616.3	956.5	17	2b2	616.3	276.1	13	2y7	616.3	827.5	21
Prostatasepzifische saure Phosphatase FVTLVFR	L	2y5	441.3	635.4	9	2b2	441.3	247.1	9	2y4	441.3	534.3	17
Prostatasepzifische saure Phosphatase FVTLVFR	H	2y5	446.3	645.4	9	2b2	446.3	247.1	9	2y4	446.3	544.3	17
Prostatasepzifische saure Phosphatase LSGLHGQDLFGIWSK	L	3y6	5553.3	737.4	9	3y13 ++	5553.3	729.4	9	3y2	5553.3	234.1	25
Prostatasepzifische saure Phosphatase LSGLHGQDLFGIWSK	H	3y6	556.0	745.4	9	3y13 ++	556.0	733.4	9	3y2	556.0	242.2	25
Vinculin ALASQLQDSLK	H	2y9	591.7	998.1	13	2y8	591.7	927.0	17	2b3	591.7	256.3	17
Vinculin GILSGTSDLLLTFDEAEVR	L	2y8	1018.5	966.5	33	3y6	679.4	718.3	21	3y8	679.4	966.5	21
Vinculin GILSGTSDLLLTFDEAEVR	H	2y8	1023.5	976.5	33	3y6	682.7	728.3	21	3y8	682.7	976.5	21
Vinculin SLGEISALTSK	L	2y9	553.6	906.0	17	2y6	553.6	606.7	13	2b2	553.6	201.2	5
Vinculin SLGEISALTSK	H	2y9	557.6	914.0	17	2y6	557.6	614.7	13	2b2	557.6	201.2	5

Supplementary table 9: Concentration, molecular weight, and purity of the measured peptides as determined with AAA and CZE. Correction factors for absolute quantification of the peptides were determined from these AAA and CZE values.

Protein / Peptide	Uniprot Accession number	molecular weight natural peptide	molecular weight SIS-peptide	used concentration [fmol]	AAA	CZE [%]	correction factor	absolute quantifiaction
Galectin-3 IALDFQR	P17931	862.0	871.5	2000	167.1	92.0	1.538	yes
Galectin-3 IQVLVEPDHFK	P17931	1324.5	1331.7	200	46.5	92.8	0.432	no
Galectin-3 VAVNDAHLLQYNHR	P17931	1649.8	1658.8	2000	91.2	99.3	0.906	no
Galectin-3 GNDVAFHFNPR	P17931	1273.4	1282.4	2000	ns	ns	ns	no
prostatic acid phosphatase (PAP) FQELESETLK	P15309	1223.4	1230.5	2000	100.0	100.0	1.000	no
prostatic acid phosphatase (PAP) FVTLVFR	P15309	881.1	890.1	2000	80.0	67.6	0.541	yes
prostatic acid phosphatase (PAP) LSGLHGQDLFGIWSK	P15309	1657.9	1665.0	200	90.0	61.3	0.552	no
Vinculin ALASQLQDSLK	P18206	1173.3	1180.6	200	ns	ns	ns	no
Vinculin GILSGTSDLLLTFDEAEVR	P18206	2036.3	2045.0	20000	ns	ns	ns	no
Vinculin SLGEISALTSK	P18206	1105.3	1112.6	2000	ns	ns	ns	yes

AAA= amino acid analysis in [%] purity; CZE= capillar zone electrophoreses in [%] purity of the peptide; ns = not specified

Supplementary table 10: 2D-DIGE analysis of protein spots differentially expressed in tumor-free and prostate cancer prostatectomy samples

Spot	Tumor		non-recurrent Pca		recurrent Pca		Tumor free		Ratio Tf vs Tu	p-value (T-Test) Tf vs Tu	p-value (U-Test) Tf vs Tu
	Mean	RSD	Mean	RSD	Mean	RSD	Mean	RSD			
down 01	200.2	126.7	215.7	80.6	183.3	165.6	4349.3	206.7	21.8	95.7	0.035
down 02	126.1	49.3	133.1	47.2	121.8	48.3	216.5	89.2	1.7	94.7	0.024
down 03	137.0	56.0	127.6	68.7	138.9	46.5	216.2	69.5	1.6	96.1	0.110
down 04	183.5	77.0	148.2	71.9	206.1	77.8	676.8	155.9	3.8	96.3	0.012
down 05	191.7	76.1	164.1	70.8	206.2	79.8	2929.6	300.9	15.7	84.4	0.024
down 06	211.8	95.3	160.8	68.4	246.6	100.7	1866.1	233.5	9.1	91.5	0.009
down 07	119.3	115.8	97.4	38.2	136.1	133.8	317.9	130.5	2.7	95.5	0.009
down 08	170.1	66.4	177.3	58.7	157.0	74.7	474.4	107.0	2.8	99.0	0.019
down 09	180.4	63.8	180.9	62.6	174.7	65.0	523.7	138.1	2.9	96.5	0.042
down 10	172.7	65.0	182.8	58.5	156.9	71.9	498.7	107.6	2.9	99.1	0.020
down 11	70.2	129.5	52.7	79.2	82.3	140.3	149.9	137.8	2.2	88.5	0.026
up 01	220.2	116.4	321.4	78.5	127.4	174.7	91.5	160.6	-2.4	89.6	0.042
up 02	1736.6	422.5	3556.3	289.7	68.5	126.2	37.2	102.7	-46.7	59.5	0.049
up 03	568.8	120.5	890.1	89.8	274.4	133.3	152.1	128.6	-3.7	96.2	0.033

PCa= prostate cancer ; RSD= relative standard deviation; - Rec = Prostate cancer without recurrence; + Rec = Prostate cancer with recurrence; tf = tumor free; tu = tumor; U-test = two-sided Mann-Whitney U-Test; mean = normalized spot volume; ratio = division of the mean.

Supplementary table 11: 2D-DIGE analysis of protein spots differentially expressed in the prostate cancer tissue from patients without relapse compared to prostate cancer tissue of patients with relapse

Spot	Tumor free		Tumor (recurrent and non recurrent)		non-recurrent Pca		recurrent Pca		Ratio + vs - Rec	p-value (T-Test) + vs - Rec	p-value (U-Test) + vs - Rec
	Mean	RSD	Mean	RSD	Mean	RSD	Mean	RSD			
down 01	100.6	96.8	587.0	332.6	1005.1	255.6	83.7	94.8	**-12.0**	73.1	0.031
down 02	25.5	41.1	40.5	153.8	59.0	133.7	16.8	76.5	**-3.5**	89.1	0.019
down 03	20.8	81.0	31.4	180.4	49.4	145.4	9.2	58.7	**-5.3**	90.8	0.023
down 04	17.3	184.5	24.5	276.0	40.0	220.9	5.4	82.3	**-7.5**	77.2	0.012
down 05	398.2	184.7	171.4	121.7	249.0	101.5	77.2	69.5	**-3.2**	95.3	1.169
down 06	107.7	195.5	118.2	97.3	162.9	79.1	63.1	94.6	**-2.6**	96.4	0.023
down 07	118.1	151.9	78.6	77.6	96.4	61.0	57.8	93.4	**-1.7**	86.7	0.036
down 08	37.3	166.8	74.8	227.9	119.2	185.2	21.4	74.5	**-5.6**	82.4	0.031
down 09	86.6	168.7	57.0	112.0	76.9	78.7	35.4	161.4	**-2.2**	87.8	0.049
down 10	27.5	113.6	16.1	63.9	20.7	52.1	10.7	56.0	**-1.9**	98.2	0.031
down 11	113.3	52.3	104.1	97.7	125.1	97.8	75.4	77.4	**-1.7**	74.5	0.036
down 12	293.0	105.8	1268.0	193.4	2029.9	148.1	228.2	177.7	**-8.9**	91.2	0.044
down 13	378.3	298.9	6149.5	416.8	11152.3	302.6	40.7	130.4	**-274.3**	66.6	0.041
down 14	184.3	243.6	134.0	111.8	186.3	87.9	79.1	124.7	**-2.4**	91.3	0.036
down 15	1530.6	336.1	86.9	145.7	114.2	134.4	49.5	139.6	**-2.3**	76.7	0.014
down 16	800.1	185.0	387.8	222.0	582.9	192.4	170.3	162.2	**-3.4**	72.8	0.033
up 01	184.3	97.5	77.6	85.1	49.8	76.1	109.5	66.6	**2.2**	97.3	0.031
up 02	91.5	160.6	202.9	122.5	127.4	174.7	321.4	78.5	**2.5**	92.5	0.049
up 03	180.3	151.9	84.3	97.8	50.3	63.6	126.4	78.0	**2.5**	97.5	0.036
up 04	100.4	138.8	124.4	180.5	46.0	119.3	226.6	127.5	**4.9**	94.5	0.027
up 05	241.6	194.7	104.2	72.3	74.7	74.7	136.2	56.8	**1.8**	95.2	0.056
up 06	352.7	269.9	511.5	325.7	141.0	228.3	896.0	253.1	**6.4**	71.2	0.042
up 07	37.2	102.7	1814.3	412.9	68.5	126.2	3556.3	289.7	**51.9**	72.5	0.031
up 08	392.3	106.8	228.3	53.3	247.5	63.3	214.3	24.3	**1.2**	47.1	0.951
up 09	197.3	75.8	213.4	151.9	124.5	82.3	324.7	129.0	**2.6**	84.2	0.045
up 10	589.4	174.3	359.2	71.9	294.8	84.9	432.5	54.3	**1.5**	79.1	0.042
up 11	126.6	59.3	255.2	82.4	163.1	66.8	524.4	105.7	**3.2**	95.3	0.042
up 12	152.1	128.6	523.7	127.2	274.4	133.3	890.1	89.8	**3.2**	96.8	0.074
up 13	699.5	111.3	1196.4	148.4	418.7	204.8	2065.4	97.9	**4.9**	97.8	0.004

PCa= prostate cancer; RSD= relative standard deviation; - Rec = Prostate cancer without recurrence; +Rec = Prostate cancer with recurrence; tf = tumor free; tu = tumor; U-test = two-sided Mann-Whitney U-Test; mean = normalized spot volume; ratio = division of the mean.

Supplementary table 12: Mass spectrometry identification of proteins differentially-expressed in tumor and tumor-free prostatectomy samples. Deregulated proteins were detected using 2D-DIGE analysis and identified using mass spectrometry

Spot	Acc. No.	Protein name	Instrument	MS				MS/MS			MW	pI
				Score	SC	Peptide	ppm	Score	SC	ppm		
down 01	P22626	Heterogeneous nuclear ribonucleoproteins A2/B1	UTX	148	42.2						37.4	9.3
down 02	P08670	Vimentin	UTX	114	37.1						53.6	4.9
down 03	P17661	Desmin	UTX	269	66.6						53.5	5.1
down 04	P17661	Desmin	UTX	325	83.8						53.5	5.1
down 05	P17661	Desmin	UTX	314	81.1						53.5	5.1
down 06	P17661	Desmin	UTX	316	83.0						53.5	5.1
down 07	P12277	Creatine kinase B-type	UTX	123	47.8						42.6	5.2
down 08	P09493	Tropomyosin alpha-1 chain	UTX	106	51.1						32.7	4.5
down 09	P09493	Tropomyosin alpha-1 chain	UTX	73	25.0						32.7	4.5
down 10	P09493	Tropomyosin alpha-1 chain	UTX	153	53.2						32.7	4.5
down 11	Q05682	Caldesmon	UTX	76	22.6						93.2	5.5
up 01	COEA1	Collagen alpha-1(XIV) chain	UTX	65	10		50				193.4	5.0
up 02	TCPA	T-complex protein 1 subunit alpha	UTX	70	19		50				60.3	6.0
up 03	Mix ANXA5	Annexin A5	UTX	74	35		80				35.9	4.6
	A1BG	Alpha-1B-glycoprotein	UTX	85	26		100	32	3	80	542.4	5.5
	P04217	Alpha-1B-glycoprotein	MaXis	156	10.9	5	2				54.2	5.5

MS= mass spectrometry; Acc.No.= Accession Number; MW = molecular weight; pI = isoelectric point; SC = sequence coverage; Score = Mascot Score; UTX = matrix assisted laser ionization time of flight mass spectrometry performed on an Ultraflex III or Ultraflextreme instrument (Bruker Daltonics, Bremen, Germany) respectively.

Supplementary table 13: Mass spectrometry identification of deregulated proteins differentially-expressed in prostatectomy samples of prostate cancer patients with and without relapse. Deregulated proteins were detected using 2D-DIGE analysis and identified using mass spectrometry

					MS				MS/MS				
Spot		Acc. No.	Protein name	Instrument	Score	SC	Pep-tide	ppm	Score	SC	ppm	MW	pI
down 01		FLNA	Filamin-A	UTX	89	13		50	56	0	50	280.6	5.7
down 01		P21333	Filamin-A	MaXis	457	6.0	15	3				280.6	5.7
down 02		P06396	Gelsolin	MaXis	348	17.1	13	2				85.6	5.9
down 03		P06396	Gelsolin	MaXis	139	6.8	6	3				85.6	5.9
down 04		O95394	Phosphoacetylglucosamine mutase	MaXis	136	8.3	5	512				59.8	5.8
down 05		PTGR2	Prostaglandin reductase 2	UTX	69	18		50	58	2	50	38.5	5.1
down 06		LMNA	Lamin-A/C	UTX	164	33		20	165	7	20	74.1	7.0
down 07		LMNA	Lamin-A/C	UTX	116	37		50				74.1	7.0
down 08		PYGB	Glycogen phosphorylase, brain form	UTX	182	39		25				96.6	6.8
down 09		P16870	Carboxypeptidase E precursor	MaXis	104	6.3	3	3				53.1	4.9
down 10		CO6A2	Collagen alpha-2(VI) chain	UTX	98	12		50				108.5	5.9
down 11		G6PD	Glucose-6-phosphate 1-dehydrogenase	UTX	128	26		50				59.2	6.8
			Glucose-6-phosphate 1-dehydrogenase	LTQ	47							59.2	6.8
down 12		SCRN1	Secernin-1	UTX	140	38		25	33	2	25	46.4	4.4
down 13		P01857	Ig gamma-1 chain C region	MaXis	91	8.2	3	3				36.1	9.4
down 14		TTC38	Tetratricopeptide repeat protein 38	UTX	79	22		30	33	2	10	52.8	5.8
down 15	Mix	MDHM	Malate dehydrogenase, mitochondrial	MALDI	124	54		80				35.5	8.9
		P51911	Calponin-1	MaXis	443	29.0	10	3				33.1	9.7
down 16		LEG3	Galectin-3	UTX	105	34		50	74	10	30	26.1	8.8
			Galectin-3	LTQ		39		8,8					26.1
up 01		VINC	Vinculin	UTX	74	14		80	91	1	25	123.7	5.3
up 02		COEA1	Collagen alpha-1(XIV) chain	UTX	65	10		50				193.4	5.0
up 03		GRP78	78 kDa glucose-regulated protein	MALDI	65	23		80				72.3	5.1
up 04		SYNEM	Synemin	UTX	82	11		80				172.7	5.0
up 05		PAPP	Prostatic acid phosphatase	UTX	73	20		120				44.5	6.2
up 06		ANXA4	Annexin A4	MALDI	58	22		30				35.9	5.8
up 07		TCPA	T-complex protein 1 subunit alpha	UTX	70	19		50				60.3	6.0
up 08	Mix	Q9UBR2	Cathepsin Z precursor	MaXis	137	9.6	3	2				33.8	6.9
		P20774	Mimecan precursor	MaXis	128	11.4	4	1				33.9	5.3
up 09		KCD12	BTB/POZ domain-containing protein KCTD12	UTX	94	43		80				35.7	5.3

up 10		CAZA1	F-actin-capping protein subunit alpha-1	UTX	114	57		50				32.9	5.5
up 11		GLO2	Hydroxyacylglutathione hydrolase, mitochondrial	UTX	62	28		50				33.8	9.3
up 12	Mix	ANXA5	Annexin A5	UTX	74	35		80				35.9	4.6
		A1BG	Alpha-1B-glycoprotein	UTX	85	26		100	32	3	80	542.4	5.5
		P04217	Alpha-1B-glycoprotein	MaXis	156	10.9	5	2				54.2	5.5
up 13		PRDX4	Peroxiredoxin-4	UTX	89	30		50				30.5	6.2
up 13		Q13162	Peroxiredoxin-4	MaXis	182	22.9	6	2				30.5	5.8

MS= mass spectrometry; Acc.No.= Accession Number; MW = molecular weight; pI = isoelectric point; SC = sequence coverage; Score = Mascot Score; UTX = matrix assisted laser ionization time of flight mass spectrometry performed on an Ultraflex III or Ultraflextrme (Bruker Daltonics, Bremen, Germany) respectively; LTQ = LTQ velos pro or LTQ oribtrap velos (Thermo Scientific, San Jose, USA) used for liquid chromatography mass spectrometry, respectively

Supplementary table 14: Secernin-1 (SCRN1) expression of the analyzed tissue microarray (TMA) of 124 tumor free tissues, 49 intraepithelial neoplasia (PIN), 16 recurrent prostate cancer patients (T + Rec) and 52 prostate cancer patients without relapse (T – Rec)

Tissue	Mean SCRN1 Score	SD SCRN1 Score	Mean SCRN1 [%] stained cells	SD SCRN1 [%] stained cells	Mean SCRN1 Remmele Score*	SD SCRN1 Remmele Score*
Tumor free	2.60	0.64	12.87	10.36	3.69	1.68
PIN	2.24	0.80	9.98	14.05	2.71	1.57
T - Rec	0.04	0.19	100.00	0.00	0.15	0.78
T + Rec	0.00	0.00	100.00	0.00	0.00	0.00

SD=standard deviation, Score = immunohistochemical score;
*adapted remmele score (from nuclear staining adapted to cytoplasmic staining)

Supplementary table 15: Vinculin expression of the analyzed tissue microarray (TMA) consisting of 125 tumor free tissues, 54 intraepithelial neoplasia (PIN), 16 recurrent prostate cancer patients (T + Rec) and 55 prostate cancer patients without relapse (T – Rec)

Tissue	Mean vinculin Score	SD vinculin Score	Mean vinculin [%] of stained cells	SD vinculin [%] of stained cells	Mean vinculin remmele score*	SD vinculin remmele score*
Tumor free	1.47	0.94	50.10	39.40	2.95	2.008
PIN	1.39	0.74	86.24	22.97	4.76	2.56
T - Rec	1.22	0.76	84.98	23.44	4.16	2.56
T + Rec	1.44	0.81	74.88	35.42	4.31	2.47

SD=standard deviation; Score = immunohistochemical score;
*adapted remmele score (from nuclear staining adapted to cytoplasmic staining)

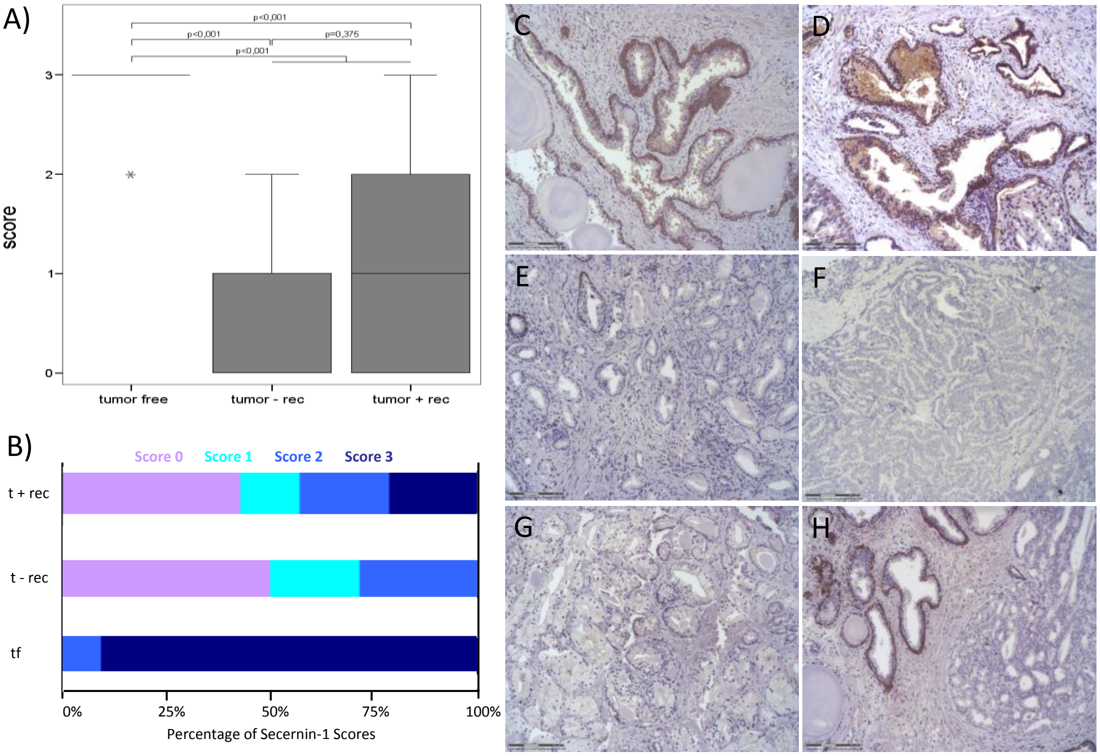

Supplementary figure1: Immunohistochemistry of secernin-1 in 13 prostatectomy samples of prostate cancer patients without relapse and 14 prostatectomy samples of prostate cancer patients with relapse as well as 27 corresponding tumor free tissues. Additionally 15 prostatectomy samples of patients with benign prostate hyperplasia were stained and analyzed. All samples were obtained from the University Hospital Dresden. **A)** Boxplots of the immunohistological scores of the stained tissue. **B)** Percentage of each sore in each analyzed patients group. **C** and **D)** Representative tumor free prostatectomy samples. **E** and **F**) Representative prostatectomy samples of prostate cancer patients without relapse. **G** and **H)** Representative prostatectomy samples of prostate cancer patients with relapse. Secernin-1 is significantly down regulated in prostate cancer specimens compared to tumor free tissue samples (p<0,001).
